# Dissecting the evolutionary role of the *Hox* gene *proboscipedia* in *Drosophila* mouthpart diversification by full locus replacement

**DOI:** 10.1126/sciadv.abk1003

**Published:** 2021-11-10

**Authors:** Ankush Auradkar, Emily A. Bulger, Sushil Devkota, William McGinnis, Ethan Bier

**Affiliations:** 1 Section of Cell and Developmental Biology, University of California San Diego, La Jolla, CA 92093, USA.; 2 Tata Institute for Genetics and Society-UCSD, La Jolla, CA 92093-0335, USA.; 3 Developmental and Stem Cell Biology Graduate Program, University of California San Francisco, and Gladstone Institutes, San Francisco, CA 94158, USA.

## Abstract

*Hox* genes determine positional codes along the head-to-tail axis. Here, we replaced the entire *Drosophila melanogaster proboscipedia* (*pb*) *Hox* locus, which controls the development of the proboscis and maxillary palps, with that from *Drosophila mimica*, a related species with highly modified mouthparts. The *D. mimica* replacement rescues most aspects of adult proboscis morphology; however, the shape and orientation of maxillary palps were modified, resembling *D. mimica* and closely related species. Expressing the *D. mimica* Pb protein in the *D. melanogaster* pattern fully rescued *D. melanogaster* morphology. However, the expression pattern directed by *D. mimica pb* cis-regulatory sequences differed from that of *D. melanogaster pb* in cells that produce altered maxillary structures, indicating that *pb* regulatory sequences can evolve in related species to alter morphology.

## Teaser

Full replacement of *D. melanogaster pb Hox* locus with *D. mimica pb* locus produces mouthparts with similarities to *D. mimica.*

## INTRODUCTION

*Hox* genes encode a family of Homeobox-containing transcription factors that specify the body plan of animals on the head-tail axis in bilateral animals (*1*–*5*). The crucial function of these genes is highlighted by the marked duplications of axial body structures, called homeotic transformations, that arise when *Hox* genes are activated in inappropriate positions during animal development (*6*). Development of head structures in the fruit fly *Drosophila melanogaster* (*D.mel*) is specified by the Hox proteins Lab, Dfd, Pb, and Scr (*7*–*9*). A fundamental question regarding *Hox* gene function is whether these genes provide permissive abstract positional codes serving primarily as regulatory scaffolds to regulate the expression patterns of differing sets of downstream genes or, alternatively, play instructive roles in controlling morphological innovations. Another central question that has vexed evolutionary geneticists is the relative importance of coding versus regulatory sequence changes in the context of evolution. The highly conserved homeodomain of Hox proteins binds DNA through a helix-turn-helix motif (*10*–*13*) and has varied little among Hox orthologs in the past 500 million years. Insect and vertebrate Hox proteins are, to some extent, interchangeable (*10*, *11*), and studies suggest that protein sequences outside the homeodomains of Lab, Pb, and Ubx orthologs have diverged to adopt new functions (*14*–*17*). *Hox* cis-regulatory elements diverge rapidly during evolution, even in closely related species, as shown in comparative studies of the *Ubx* and *Abd-B Hox* genes in drosophilids (*18*–*20*).

**Fig. 1.**
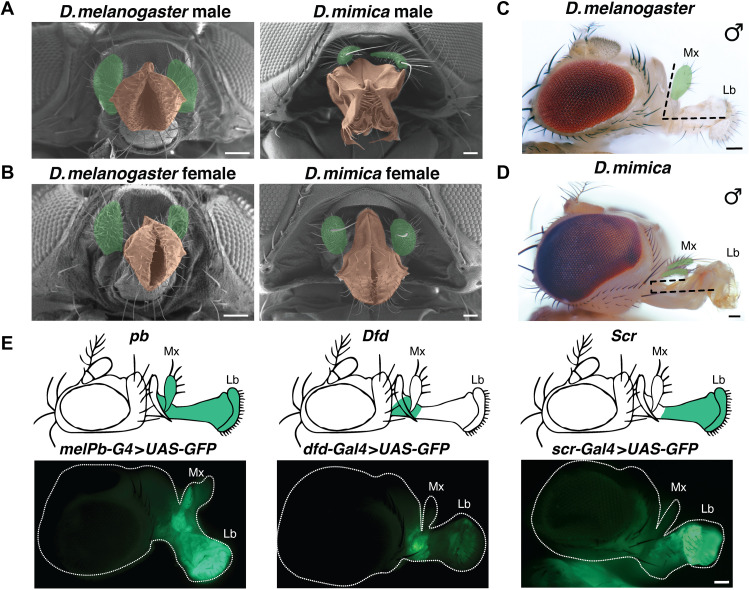
Mouthparts of *D.mel* and *D.mim* and *Hox* gene expression in maxillary and labellar region. (**A**) Scanning electron microscopy (SEM) image of *D.mel* and *D.mim* male adult head with mouthparts. (**B**) SEM image of *D.mel* and *D.mim* female adult head with mouthparts. False color was assigned to highlight maxillary palps (green) and labellar lobes (tan). (**C**) Bright-field image of the *D.mel* head. Dotted lines highlight the perpendicular orientation of maxillary palps (Mx) relative to the labellum (Lb). (**D**) Bright-field image of the *D.mim* head. Dashed lines indicate the parallel orientation of the maxillary palps and labellum. (**E**) Top: Schematic representation of *Hox* gene expression patterns for *pb*, *Dfd*, and *Scr* in *D.mel.* Bottom: Gal4-tagged *pb*, *Dfd*, and *Scr* driving expression of *UAS-nlsGFP* in adult mouthparts. Scale bars, 50 μm.

The *Drosophila pb* locus, which specifies head structures including the mouthparts, provides an excellent system for examining central questions of *Hox* gene evolution. These structures are morphologically quite distinct in two species of *Drosophila—D.mel and D. mimica* (*D.mim*)—and are sexually dimorphic in *D.mim*. Males of *D.mim* have highly modified mouthparts with the labellum consisting of enlarged spine-like setae (Fig. 1A) (*21*, *22*), while females of this species resemble monomorphic *D.mel* flies (Fig. 1B). During development, the maxillary palps are derived from maxillary anlagen in the eye-antennal imaginal discs, while the proboscis derives from labial discs. *pb*-null mutants are viable (which is not true for other fly Hox loci) and develop prothoracic legs in place of labial palps (*23*). Hypomorphic alleles of *pb* show a transformation of labial palps to the aristal segment of the antennae (*7*). Crucially, the *pb* locus is of a manageable size (~38 kb) for genetic recombineering and can be rescued by a *D.mel pb* minimal transgene (*24*). Because of these attributes, we chose *pb* as a model gene to perform replacement studies. The recent advent of highly efficient CRISPR-Cas9 genome editing tools has provided the means to precisely substitute the entire *pb* gene locus and observe the resulting effects on development of mouthpart structures in adult flies (in an otherwise *D.mel* background). This approach enables a rigorous decoding of newly evolved *Hox* gene functions.

## RESULTS AND DISCUSSION

### Sex-specific modification of mouthparts in *D.mim*

The Drosophilidae encompass a diverse family of flies exhibiting a wide variety of morphological and ecological adaptations as exemplified by drosophilids endemic to the Hawaiian Islands. Among the Hawaiian flies, the modified mouthpart species group includes ~86 described species (*22*), one of which, *D.mim*, displays a particularly pronounced sexual dimorphism. This species is poorly studied as there has been little success in long-term maintenance of this species in stock centers or laboratory colonies. Because of its distinctive mouthparts and moderate phylogenetic distance from the standard laboratory species *D.mel*, we endeavored to overcome these obstacles. We collected *D.mim* from the Hawaii Volcanoes National Park, established conditions for maintaining and propagating this species in the laboratory for a period of over 1 year, sequenced the *D.mim* genome using a combination of short (Illumina) and long (Sanger) reads, and assembled a high-quality contig spanning the *pb* locus (data S1).

**Fig. 2.**
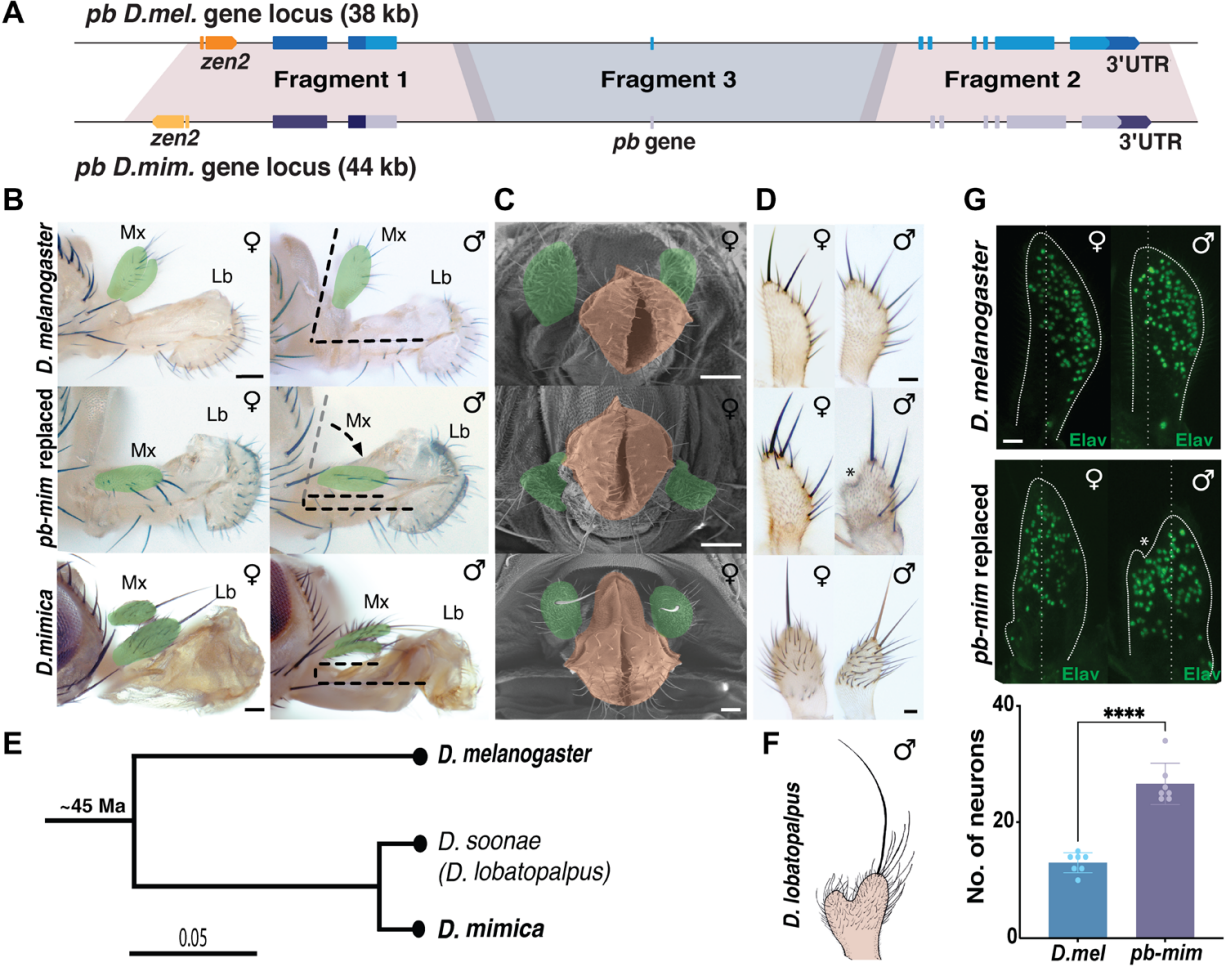
Replacement of *D.mel pb* genomic locus with that of *D.mim.* (**A**) Diagram of the *pb* genomic locus from *D.mel* (38 kb) and *D.mim* (44 kb)*. pb*-Fragments 1, 2, and 3 were sequentially replaced to generate a full locus replacement with *pb-mim* sequences. (**B**) Bright-field image of female and male heads. The dashed lines highlight the relative orientations of maxillary palps and labellum in *D.mel*, *pb-mim* replaced, and *D.mim.* False color highlights the maxillary palps (green). (**C**) SEM image of *D.mel*, *pb-mim* replaced, and *D.mim* female adult heads with mouthparts. False colors were also assigned to highlight the maxillary palp (green) and the labellar lobes (tan). Scale bars, 50 μm. (**D**) Female and male maxillary palps of *D.mel*, *pb-mim* replaced, and *D.mim.* Scale bars, 25 μm. Asterisks here and in panel G indicate notch on the inner surface. (**E**) Phylogenetic tree of three *Drosophila* species based on conserved amino acids based on the maximum likelihood method using the CLC Main Workbench program. Bars indicate evolutionary distance. (**F**) Sketch of a *D. lobatopalpus* male maxillary palp (provided by S. Roy). (**G**) For *D.mel* and *pb-mim* replaced, the neurons of the female and male maxillary palps were stained with anti-Elav antibodies. Dotted white lines denote the boundaries of the maxillary palps. Scale bars, 25 μm. The graph at the bottom shows the number of neurons on the medial surfaces (left side of dotted line) of maxillary palps in *D.mel* and *pb-mim* replaced flies. Error bars indicate SD; number of maxillary palps scored (*n*) = 7. ****P < 0.0001.

*D.mim* males display striking sex-specific modifications of the proboscis and labellar lobes. The labellar rim is significantly modified in shape (Fig. 1A). The most dorsal portion of the rim is bordered by a fringe of thin setae, and the ventral setae are enlarged into large, heavily sclerotized tusk-like spines. In contrast, the labellar lobes in females are morphologically similar to those of *D.mel* (Fig. 1B). In addition, the maxillary palps are modified in shape, distribution of setae, and orientation relative to the proboscis. In *D.mim* males, palps are rounded at the apex with a single long bristle at the apex and thinner setae along the dorsal and ventral margins (Figs. 1A and 2D), while, in females, palps are sharply pointed with a single prominent apical bristle and thick, dense setae on dorsal and ventral surfaces (Figs. 1B and 2D). Further, in *D.mim*, the palps in both sexes lie parallel to the proboscis, whereas in *D.mel* these organs are oriented perpendicular to its long axis (Fig. 1, C and D).

### Expression of *pb*, *Dfd*, and *Scr* in developing *D.mel* mouthparts

Three adjacent *Hox* genes—*pb*, *Dfd*, and *Scr*—collaborate to specify the mouthparts (*7*–*9*). As a first step in understanding the overlapping role of these *Hox* genes in diversification of drosophilid mouthparts, we investigated their expression patterns in *D.mel* by tagging them with a *GAL4-T2A* cassette (Fig. 1E). The *GAL4-T2A* sequences were inserted 5′ to the translation initiation codons of each of the *Hox* genes to generate fusion transcripts that are in-frame with the endogenous genes. These *GAL4-T2A* insertions preserve expression and activity of the tagged *Hox* loci while also producing a separate GAL4 transactivating protein (due to ribosome skipping) expressed in the same pattern. These engineered strains can be crossed to a *UAS-nlsGFP* strain to perform live imaging of *Hox* expression patterns in larvae and adults (figs. S1 and S2).

Movies of *pb-GAL4>UAS-GFP* individuals revealed strong green fluorescent protein (GFP) reporter gene expression in the salivary glands, brain, and labial imaginal discs (fig. S2A). Continuous GFP expression during differentiation of the mouthparts (fig. S2A and movie S1) initiates just before head eversion (−4 to +6 hours after head eversion) in a broad band of cells, giving rise to the ventrally oriented proboscis. This expression territory then separates into symmetric dorsal-anterior subdomains corresponding to anlagen of the maxillary palps from ~9 to 56 hours after head eversion (fig. S2A), resulting in a stable pattern of GFP expression in the maxillary socket, anterior projecting maxillary palps, and the elongated downward-facing proboscis (Fig. 1E). In *D.mel* adults, the perpendicular orientation of the maxillary palps relative to the proboscis is readily apparent in frontal (Fig. 1, A and B) and lateral (Fig. 1C) views of adult *D.mel* mouthparts.

Because neighboring *Hox* genes often engage in cross-regulatory interactions, we more closely examined expression of *Dfd*, which is also required for maxillary palp development (fig. S2B and movie S2) (*8*, *25*). *Dfd-GAL4*–driven GFP expression, observed first in the ventral nerve cord and eye-antennal imaginal discs (fig. S2B), then intensifies in specific domains of the palatal plate, giving rise to the maxillary socket, which lies at the base of the maxillary palp and later in a narrow band of cells comprising the distal labellum of the proboscis (fig. S2B). Notably, expression of *Dfd* and *pb* overlaps in cells, giving rise to the maxillary socket, which serves as the point of juncture between the maxillary palps and the proboscis (fig. S2, A and B).

*Scr* also has been reported to be expressed in the labial disc and is required for formation of the proboscis (*26*). Consistent with these prior observations, *Scr-GAL4* drives GFP expression in a dynamic pattern during pupal metamorphosis (fig. S2B and movie S3) and, in adults, becomes restricted to the proboscis and excluded from the maxillary palps (Fig. 1E).

### Replacement of the *D.mel pb* locus with orthologous sequences from *D.mim*

As a first step in replacing the *D.mel pb* locus with orthologous sequences from *D.mim*, we deleted the entire *D.mel* locus and replaced it with a GFP marker cassette using CRISPR-Cas9–assisted homology-directed repair (fig. S3A). Flies homozygous for this complete *pb* locus deletion were viable, with the labial palps partially transformed into first thoracic legs and maxillary palps of reduced in size (fig. S3B), as previously reported for *pb*-null mutants (*7*, *25*). On the basis of our genomic sequence of the *D.mim pb* locus, we assembled three overlapping *D.mim* cassettes spanning ~44 kb, which we then iteratively inserted precisely into the *D.mel pb* locus using CRISPR-Cas9–directed genome cleavage and homology-directed recombination (Fig. 2A, Supplementary Text, and figs. S4 and S5).

Three independently isolated transformant lines with the entire *D.mel pb* locus replaced with that from *pb mimica* were recovered, which all manifested the same phenotypes described below. The structure of the entire replaced *pb* locus was verified by DNA sequencing in one of these lines, which we refer to as the *pb-mim* line hereafter. Homozygous *pb-mim* replacement flies were viable and fertile, and most of their mouthpart structures were indistinguishable from those of wild-type *D.mel*. In particular, the labellar lobes, which are dimorphic in *D.mim* males, displayed the standard *D.mel* morphology in both sexes of *pb-mim* lines (Fig. 2, B and C). However, in *pb-mim* males and females, the maxillary palps were oriented parallel to the proboscis (Fig. 2B), in contrast to their orthogonal arrangement in wild-type *D.mel* (Fig. 2, B and C). This parallel alignment of the maxillary palps and proboscis in *pb-mim* replacement flies resembles that of both *D.mim* sexes (Fig. 2, B and C). We also observed an altered shape of the maxillary palps in *pb-mim* replaced flies. The palps were particularly altered in males, which were bulbous with a shallow notch on the inner surface (Fig. 2D and fig. S6). Although this modified morphology of the maxillary palps does not resemble that of either *D.mel* or *D.mim*, it is characteristic of the same organ in two close Hawaiian relatives of *D.mim*—*D. soonae* and *D. lobatopalpus* (Fig. 2E). In these latter species, males display flat palps with a single hook-like large notch (redrawn in Fig. 2F) (*22*), which may be an ancestral phenotype of this Hawaiian clade. We also observed increased numbers of underlying sensory neurons in the *pb-mim* replacement, which were distributed more broadly throughout the palp than in *D.mel* (Fig. 2G).

### Pb protein function has been conserved in mouthpart specification

**Fig. 3.**
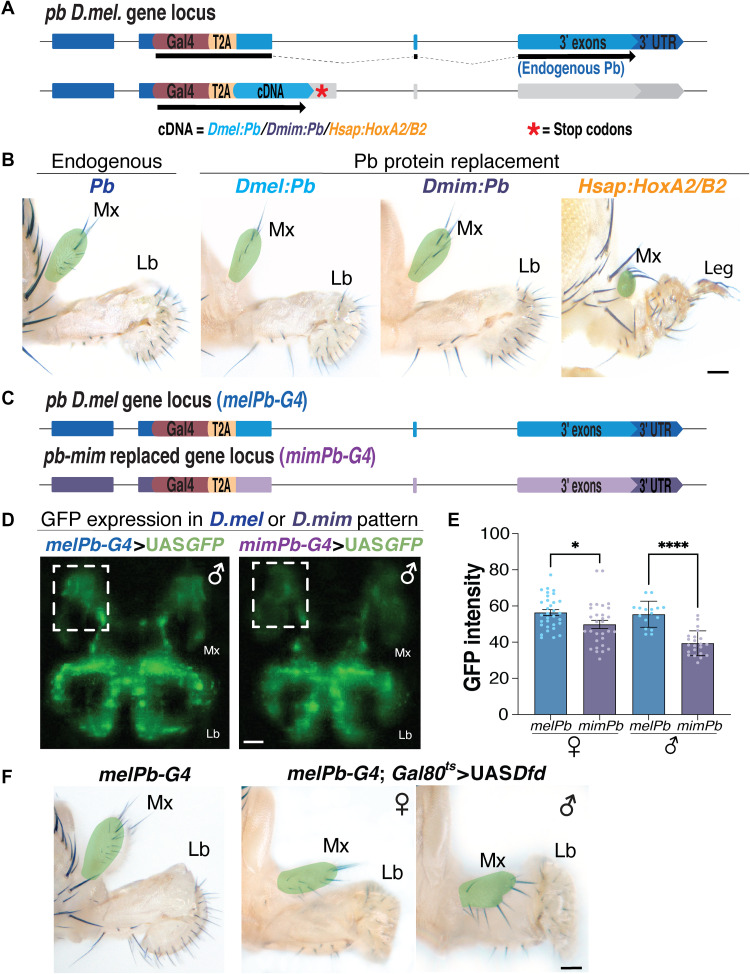
*pb* expression pattern changes in concert with changes in maxillary palp structure and orientation in *pb-mim* replaced flies. (**A**) Schematic of the *pb* genomic region incorporating a *Gal4-T2A* insertion at the *pb* start codon (endogenous) followed by the cDNAs encoding *D.mel:Pb*, *D.mim:Pb*, or the human *Hox pb* ortholog (*H.sap:HoxA2*) with transcription terminated by two stop codons. (**B**) Homozygous endogenous *Pb*, *D.mel:Pb* cDNA, *D.mim:Pb* cDNA, and *H.sap:HoxA2* cDNA fly heads. Scale bar, 50 μm. (**C**) Schematic of the *pb D.mel* genomic region with a *Gal4-T2A* insertion at the *pb* start codon (*melPb-G4*, top) and the *pb-mim* replaced genomic region with a *Gal4-T2A* insertion at the *pb* start codon (*mimPb-G4*, bottom). (**D**) Expression patterns provided by *melPb-G4* and *mimPb-G4* are shown as marked by their activation of *UAS-nlsGFP* in maxillary palps and proboscis in late pupae; the dashed box indicates the region of Pb expression changed between *melPb-G4* and *mimPb-G4*. Scale bar, 50 μm. (**E**) Graph represents normalized mean GFP intensity between dashed box region with labellum in *melPb-G4* and *mimPb-G4* female (*n* = 30) and male (*n* = 22) late pupae. Error bars indicate SD. *P < 0.05, and ****P < 0.0001. (**F**) Representative bright-field image of heads of flies with either *melPb-G4* controls (left) or *melPb-G4*>*UAS-Dfd* with *tub-Gal80^ts^* female (center) and male (right) head. Scale bar, 50 μm.

The differences in maxillary palp orientation and shape observed in the *pb-mim* replacement strain could be due to changes in Pb protein function or might reflect alterations to cis-regulatory elements controlling *pb* transcription. Over the period of ~45 million years since the separation of the *D.mel* and *D.mim* species, their Pb protein sequences have diverged 34.7%, which is considerably greater than that observed within the more closely related Hawaiian clade (fig. S7, A to D). We examined the potential role this substantial sequence divergence might play in the altered orientation and shape of maxillary palps in the *pb-mim* replacement by expressing a complementary DNA (cDNA) encoding the *D.mim* Pb protein under the control of *D.mel* regulatory sequences. We used the same locus-tagging strategy as discussed above for integrating Gal4-T2A-cDNA cassettes into noncoding sequences of the first exon of the *D.mel pb* locus (Fig. 3A and fig. S7E). These Gal4-T2A-cDNA cassettes, however, also contained two stop codons at the end of the cDNA coding sequence to terminate translation. Homozygous strains for this *Gal4-T2A-pbmim* insertion express Pb protein from the inserted construct under the control of *D.mel* regulatory elements while eliminating endogenous Pb protein expression. As expected, flies expressing the *D.mel* Pb protein under control of *D.mel* regulatory sequences developed mouthparts that were indistinguishable from those of *D.mel* (Fig. 3B, second panel). Similarly, expression of a cDNA encoding the *D.mim.* Pb protein provided full rescue of *pb* function (Fig. 3B, third panel). In contrast, expression of cDNAs encoding either of the two human Pb Hox protein orthologs (*GAL4-T2A-HoxA2/B2)* did not rescue Pb function, exhibiting null loss-of-function phenotypes in which prothoracic legs formed in the place of labial palps, indicating that human HoxA2/B2 proteins expressed in the *D.mel* pattern cannot substitute for endogenous Pb activity (Fig. 3B, fourth panel, and fig. S7F).

### Differential regulation of *mel-pb* versus *mim-pb* in pupal maxillary palp primordia

Comparison of *Pb* genomic sequences from the three *Drosophilid* species *D.mel*, *D.grim*, *and D.mim* locus again revealed a much higher degree of nucleotide sequence similarity over the entire length of the locus between *D.mim* and its more closely related cousin *D.grim* than between either of these species and *D.mel*, consistent with inferred phylogenetic relationships (fig. S8, A and B). We tested whether this extensive distributed sequence divergence, which also encompasses noncoding sequences, resulted in different patterns of GFP expression driven by *GAL4* driver cassettes inserted into the *D.mel pb* versus *D.mim pb* loci (Fig. 3C). As the morphological development of adult mouthparts occurs primarily during pupal stages, we focused our attention on imaging live pupae spanning this period of approximately 60 hours (Fig. 3D). Notably, there was significantly lower GFP intensity in the maxillary cap primordia in *pb*-*mim* replaced pupae relative to *D.mel* at comparable development stages (Fig. 3, D and E). This localized reduction was quantified in specified sectors of living pupae including the maxillary and labellar anlage, which confirmed a significant decrease in relative GFP expression in the maxillary regions compared to the labellum in the *pb-mim* replacement versus *D.mel* (Fig. 3E and fig. S8C). In contrast, we observed no significant changes in GFP intensity and expression pattern in the labellum region between the *pb-mim* replacement and *D.mel* strains (fig. S8C). These changes in relative maxillary GFP expression pattern were most evident in male pupae of the *D.mim pb* line, consistent with the sex-specific morphological changes associated with the *pb-mim* replacement (Fig. 3E).

### Ectopic expression of *Dfd* changes maxillary palp orientation

As described above, we observed significantly reduced *pb-GAL4*–driven GFP expression in the *pb-mim* replacement in primordia of the maxillary palps in regions of overlapping expression, with *Dfd* giving rise to the base of the palp (the palp socket). In *Drosophila* and other metazoans, Hox proteins often engage in cross-regulatory repressive interactions following a general rule referred to as “posterior dominance,” wherein more posterior genes exert dominance over the function of anterior orthologs in overlapping areas of expression (*27*). We therefore hypothesized that the decrease in *pb* expression in *pb-mim* might be due to altered regulatory interactions between Pb versus Dfd, thus leading to changes in maxillary palp orientation and structure resulting from a greater relative influence of Dfd.

We tested this hypothesis by overexpressing the *D.mel Dfd* protein in the normal *Pb* expression domain using the *D.mel pb-GAL4* driver. This ectopic *Dfd* expression, which included cells giving rise to the maxillary socket, resulted in pleiotropic phenotypes consisting of bilobed maxillary palps and transformation of labellar lobes into maxillary palp-like structures (fig. S9, A and B). We then restricted *Dfd* overexpression to the relevant developmental window of ~24 hours after pupae formation by combining the *melPb-GAL4* driver with a temperature-sensitive Gal80 source, which represses GAL4 activity at the permissive temperature (22°C) but permits GAL4 activity at the nonpermissive temperature (29°C). Increased *Dfd* expression confined to this narrow pupal time window resulted in more selective phenotypes that included reorientation of the maxillary palps relative to the proboscis, phenocopying the *Pb*-*mim* replaced flies (Fig. 3F). These results support the hypothesis that a regulatory relationship between Dfd and Pb plays a role in directing the parallel orientation of the maxillary palps with the axis of the proboscis, presumably by altering developmental fates, cell proliferation, or migratory behaviors of cells forming the maxillary socket.

## DISCUSSION

**Fig. 4.**
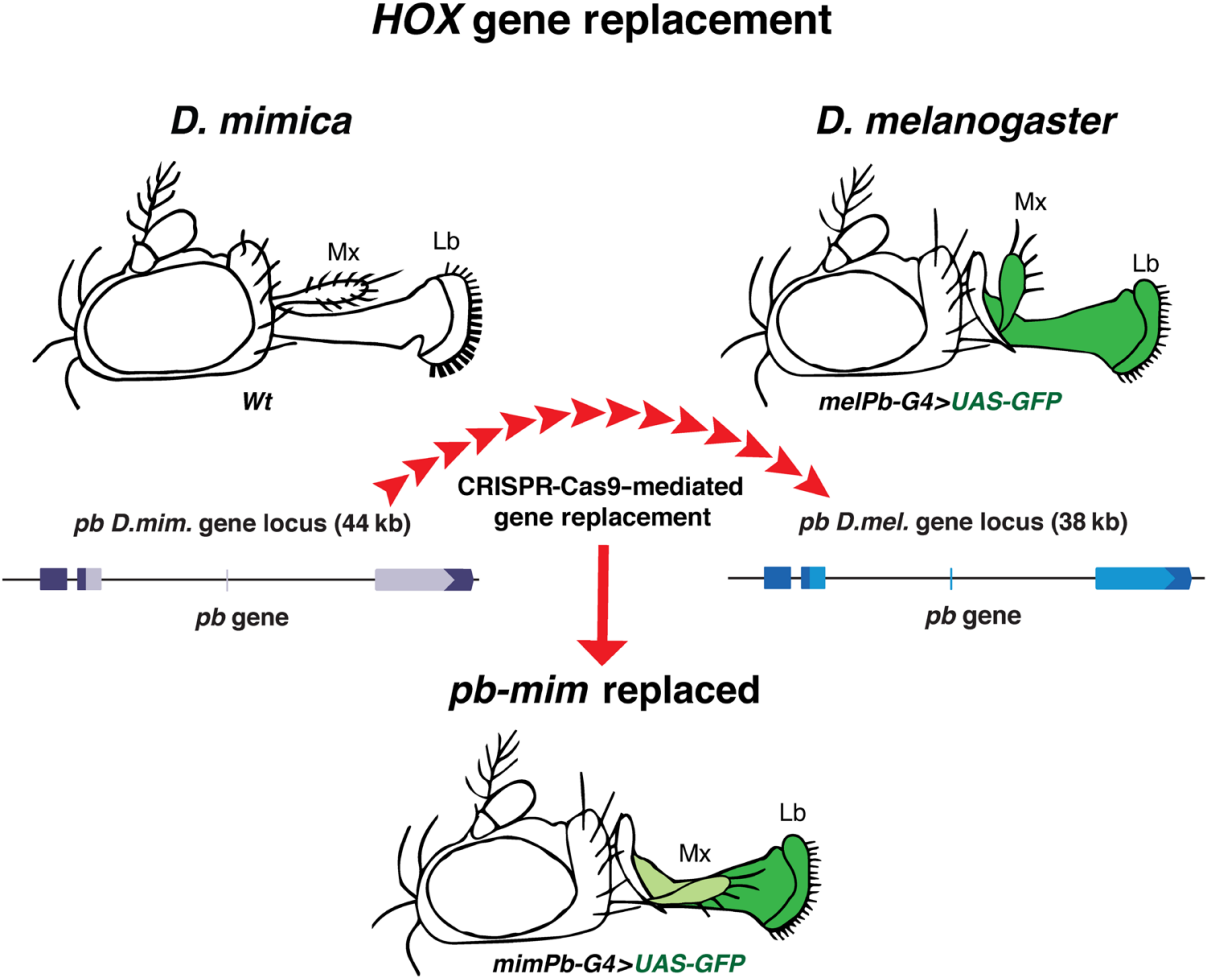
Summary diagram. Diagram illustrates full replacement of the endogenous *pb* locus of *D.mel* (fly head and gene locus on right) with orthologous sequences from *D.mim* (fly head and gene locus on left) and the effects of this replacement on adult mouthpart structures and *pb* expression (bottom).

In this study, we used CRISPR-Cas9 to generate a full replacement of the endogenous *pb* locus of *D.mel* with orthologous sequences from *D.mim* and analyzed the phenotypic effects of this replacement on adult mouthpart structures (Fig. 4). Strains carrying the full *D.mim pb* (*pb-mim*) replacement were fully viable and fertile, rescuing the endogenous morphology of the proboscis, which is deleted in *pb*^−^ loss-of-function mutants. In contrast, the shape and orientation of the maxillary palps were similar to those of *D.mim* or of its closely related species. These altered morphological features in the *pb-mim* replacement strain could be attributed to alterations in the pattern of *pb* expression rather than protein function. In particular, *pb* expression in pupal anlagen of the maxillary palps was significantly reduced relative to that in *D.mel*, potentially shifting the relative activity balance of *pb* and the competing *Hox* gene *Dfd*, which is coexpressed with *pb* in these cells. Consistent with this possibility, overexpression of *Dfd* in maxillary primordia of *D.mel* produces a phenotype very similar to that of the *pb-mim* replacement.

Our findings suggest that the *pb* locus in the two compared drosophilid species not only acts largely to provide a permissive positional code (as seen by *D.mim pb* rescuing a *D.mel pb* deletion mutant to a nearly wild-type *D.mel* pattern) but also plays an instructive role in determining the orientation and shape of the maxillary palps, which are paddle shaped and oriented perpendicular to the proboscis in *D.mel* versus heart-shaped and arranged parallel to the proboscis in male flies carrying the *D.mim pb* replacement. The heart shape of the maxillary palps in males is characteristic of neither *D.mel* nor *D.mim* but is highly similar to that found in two closely related sibling species of *D.mim*, suggesting that this trait may have been present in the common ancestor of that Hawaiian clade.

There are many differences in both the protein and noncoding sequences between *D.mel* and *D.mim* that could be responsible for the distinct instructive functions of *pb* in determining the orientation and shape of the maxillary palps. Experiments in which the protein-coding cDNAs from *D.mel* or *D.mim* were placed under the control of endogenous *D.mel* regulatory sequences revealed that these divergent proteins have retained a conserved function over the ~45 million years since the evolutionary divergence of the two species. At a much larger phylogenetic distance, however, the human cognate cDNAs encoding the HoxA2 and HoxB2 proteins failed to substitute for Pb function. This lack of activity may be attributable to the absence of a C-terminal tail in the human HoxA2/B2 proteins that is present in the Drosophilid Pb proteins, which have been shown to be essential for interaction with the neighboring Hox protein Scr (*28*, *29*).

Conservation of Pb protein function between *D.mel* and *D.mim* suggests that cis-regulatory changes underlie the maxillary palp phenotypes observed in the *D.mim pb* replacement. During the first 30 hours of pupal development, the mouthparts undergo major morphogenic events including cell migration and tissue reorganization. During this stage of pupal development, we observed significantly lower levels of GFP expression in cells contributing to the maxillary socket in the *D.mim pb* replacement relative to *D.mel*, which overlaps with expression of the *Dfd Hox* gene that may oppose Pb activity. Consistent with this hypothesis, overexpression of Dfd in *D.mel pb* pattern produced a phenocopy of the *D.mim* replacement.

With regard to Hox regulatory element evolution and morphological changes, our results add to the pioneering studies of Stern (*19*), who found that subtle changes in the expression pattern of the Ubx *Hox* gene were associated with a change in the trichome pattern between *D.mel* and *D. simulans* legs (*19*). Our analysis using an entire precise Hox gene replacement provide rigorous evidence that *Hox* gene cis-regulatory changes can result in morphological diversification in closely related drosophilids. Future studies to unravel the nature of the changed cis-regulatory sequences, and the cross-regulatory interactions between Pb and Dfd, and perhaps also with Scr, should delineate the precise mechanisms by which modest changes in gene expression can lead to the elaboration of new morphological forms.

## MATERIALS AND METHODS

### *Drosophila* rearing and fly stocks

Stocks were maintained at 18°C, while crosses were carried out at 25°C on a 12-hour day/night cycle on corn meal medium. Fly stocks used in this study are listed in table S1.

### Field collections and rearing of Hawaiian *Drosophila*

Field collections of Hawaiian *D.mim* were conducted under the U.S. Department of Interior National Park Service, scientific research and collection permits HAVO-2019-SCI-0048. Collections were made at the Kipuka Puaulu, Kipuka Ki, and Olaa Small Tract (Pole 44) in Hawaii Volcanoes National Park. Flies were collected by using disinfected aspirators and net by sweeping over *Sapindus saponaria* (Hawaiian soapberry) leaves and fruit in the national park. To simulate the cooler temperatures found in the Hawaiian rainforest, field-collected flies were transported in an ice chest to the laboratory rearing facility. Once in the laboratory, flies were transferred to wide-mouth jars filled with moist, sterilized sand. A piece of muslin cloth was placed over the mouth of the jar and held in place with the rubber band. All flies were reared at 18°C with a controlled night-day light schedule and around 50% humidity. *D.mim* were reared on Wheeler-Clayton food (*30*), Formula 4-24 instant *Drosophila* medium, and fermenting fruit of *Sapindus* supplemented every 10 days. Adults were transferred every 4 to 5 weeks into fresh jar.

### Whole-genome extraction and sequencing

Around 10 non-isogenic adult flies were used for genomic DNA preparation according to the manufacturer’s instructions of the Qiagen DNeasy Blood and Tissue Kit. Sequencing libraries were constructed using the Illumina TruSeq DNA PCR-Free Kit for 350–base pair (bp) inserts, and libraries were clustered on Illumina PE150 platform, generating 150-bp paired-end reads. Before assembly, read quality was explored using FastQC (v. 0.11.2) (*31*). Adapter-trimmed reads were aligned to the assemblies using Bowtie 2 (*32*). *D.mim* genome was aligned to the *D.mel* genome to identify conservation [National Center for Biotechnology Information (NCBI) Assembly ID: 202931, Release 6 plus ISO1 MT/UCSC Genome Browser Assembly ID: dm6] (*33*, *34*). Raw sequencing data were uploaded to the NCBI Sequence Read Archive (www.ncbi.nlm.nih.gov/bioproject/PRJNA727426).

### Plasmid construction

Homologous arms for each locus were amplified from the *w*^1118^ wild-type genomic DNA, and *D.mim pb* locus was amplified from wild-type genomic DNA using Q5 Hot Start Master Mix (New England Biolabs, catalog no. M0494S) and Gibson assembled with NEBuilder HiFi DNA Assembly Master Mix (New England Biolabs, catalog no. E2621). Full sequences of guide RNAs (gRNAs) and oligos can be found in tables S1 to S3. Plasmid sequence data have been deposited on Mendeley Data (https://data.mendeley.com/datasets/xk59988f7z/2).

### Transgenic fly construction

Transgenic flies were constructed by injecting the *D.mim pb* plasmids in nos-Cas9 attP40 fly background from Rainbow Transgenic Flies Inc. Injected embryos were received, and emerged F0 flies were crossed to *w*^1118^ flies.

Transgenic flies carrying full replacement of the *D.mel pb* genomic locus (38.5 kb) with that from *D.mim* (44 kb) were conducted in three sequential steps. Initially fragment 1, 5′ pb sequence (10 kb) was replaced with 12-kb *D.mim* 5′ fragment. Two single-guide RNAs (sgRNAs) were designed to specifically target 5′ pb sequence (10 kb) (fig. S3A). Using *nos-Cas9*, we inserted orthologous *D.mim pb* sequences, along with an eye-directed GFP (*3xP3-GFP*) marker cassette flanked by lox sites upstream of the 5′ fragment. Similarly, 3′ *D.mel pb* sequences (9.5 kb) were replaced with 14-kb *D.mim* fragment 2 along with eye-directed red fluorescent protein (RFP) marker cassette flanked by lox sites upstream of the fragment (fig. S4A). Last, the remaining middle section of the *D.mel pb* locus was replaced with 20-kb *D.mim* fragment 3. Full-replacement recombinants were identified by loss of the RFP marker cassette (Fig. 2A). Correct integration of the transgene was confirmed by polymerase chain reaction amplification, as well as through sequencing of the whole construct using primers spanning the entire *D.mim pb* locus and junction fragments within the *D.mel* genome lying outside the region that served as homology arms for integration of fragments 1 and 2.

Gal4 locus-tagged transgenic flies were constructed using standard homology-based CRISPR-Cas9 genome editing methods as previously described (*35*), with the following modifications. sgRNA constructs were generated in pCFD3 plasmid (Addgene, plasmid no. 49410; RRID:Addgene_49410) expressing pbGal4-gRNA, DfdGal4-gRNA, and ScrGal4-gRNA targeting initiation codons of each of the *Hox* genes. Donor constructs were generated with homology arms upstream and downstream of the gRNA target site. The homology arms and Gal4-T2A cassettes were combined by Gibson assembly. All injections to generate transgenic flies were performed by Rainbow Transgenic Flies Inc. Donor and sgRNA constructs were injected in nos-Cas9 attP40 flies, and emerged F0 flies were crossed with *UAS-nlsGFP* (2nd Chr, RRID:BDSC_4775) to select for the transformants.

### SEM imaging

Flies were fixed and dehydrated by immersion in increasing concentrations of ethanol in water (25, 50, 75, and 100%) 1 hour each. The samples were then dehydrated using increasing concentrations of hexamethyldisilazane in ethanol (50 and 75%, and twice in 100%) 2 hours each. The samples were air-dried for 1 hour, placed on stubs, and coated with gold. The specimens were examined with a scanning electron microscope (SEM; JEOL model JSM-5610LV).

### Immunostaining

Adult maxillary palps were dissected and immunoassayed as previously described (*36*). Primary antibody mouse anti-ELAV was used as a neuronal marker (Elav-9F8A9) (*37*) at a 1:10 dilution. Secondary antibodies used for visualization were goat anti-rabbit Alexa Fluor 488 (Invitrogen) at a 1:500 dilution and goat anti-mouse Alexa Fluor 488 (Invitrogen) at a 1:500 dilution. Slides were viewed on a Leica SP8 confocal microscope. Images were generated and analyzed using Fiji.

### Live imaging

Live samples were collected at the white pupal stage and rinsed with water to remove the fly food from their surface. Prepupae were positioned dorsal side down on a glass bottom dish. A wet tissue was kept around the specimens to maintain humidity levels during imaging. Time-lapse image acquisition was carried out for 3 to 5 days at 20-min intervals on a Zeiss fluorescence microscope. Fiji was used to generate images and measure GFP fluorescence intensity in the maxillary cap regions and labellum.

### Graph generation and statistical analysis

We used GraphPad Prism 9 to generate all our graphs. For statistical analysis, we used GraphPad Prism 9. We used unpaired *t* test to compare fluorescence intensity and to compare the number of Elav neurons.

## Supplementary Materials

### This PDF file includes:

Supplementary Text Figs. S1 to S9 Tables S1 to S7 Legends for movies S1 to S3 Legend for data S1

## Notes

View/request a protocol for this paper from *Bio-protocol*.
