# Supplementary material for: Dissecting the evolutionary role of the Hox gene proboscipedia in Drosophila mouthpart diversification by full locus replacement
Source: Sci Adv. 2021 Nov 10;7(46):eabk1003. doi: 10.1126/sciadv.abk1003 (PMC8580299; doi:10.1126/sciadv.abk1003)
Supplement: Supplementary file 1 — Supplementary Text Figs. S1 to S9 Tables S1 to S7 Legends for movies S1 to S3 Legend for data S1 [file sciadv.abk1003_sm.pdf]

Science Advances
AAAS

Supplementary Materials for

Dissecting the evolutionary role of the *Hox* gene *proboscipedia* in *Drosophila* mouthpart diversification by full locus replacement

Ankush Auradkar, Emily A. Bulger, Sushil Devkota, William McGinnis*, Ethan Bier*

*Corresponding author. Email: wmcginnis@ucsd.edu (W.M.); ebier@ucsd.edu (E.B.)

Published 10 November 2021, *Sci. Adv.* **7**, eabk1003 (2021)
DOI: 10.1126/sciadv.abk1003

The PDF file includes:

Supplementary Text
Figs. S1 to S9
Tables S1 to S7
Legends for movies S1 to S3
Legend for data S1

Other Supplementary Material for this manuscript includes the following:

Movies S1 to S3
Data S1

Supplementary Text

Results

Phenotypes of intermediate constructs

Homozygous replacement of only the 5' portion of the *D.mel pb* locus with sequences from *D.mim* (fragment 1) resulted in flies with a completely normal labellum, but with notable alterations in maxillary palp morphology and bristle patterning (fig. S4). Maxillary palps of fragment 1 replaced flies were bulbous with increased numbers of tufted bristles in the distal region of the maxillary palps (fig. S4A). Correspondingly, we observed greater numbers of underlying ELAV positive staining neurons with a broader distribution in developing pupal maxillary palps (fig. S4D), a phenotype reminiscent of that observed for the full *pb-mim* replacement (Fig. 2G). Next, we replaced the 3'-most portion of the pb locus (fragment 2) alone (fig. S5, A and B) or in combination with replacement of fragment 1 (fig. S5, C and D). Flies homozygous for fragment 2 only displayed a partial loss of the *pb* homeotic phenotype which consists of a transformation of labellum into arista (fig. S5, A and B). The hypomorphic fragment 2 phenotype dominated when combined with the fragment 1 replacement. In light of the fact that a replacement of the entire *D.mel pb* locus with that of *D.mim.* provides nearly full rescue of *pb* function, we speculate that long-range interactions between multiple species-specific cis-acting regulatory sequences are required to act in concert to provide full activity of the locus.

It is noteworthy that the phenotype of the full *D.mim pb* replacement differs from the sum of its component parts in that replacement of only 5' portion of the locus leads to alteration in bristle numbers and underlying neurons (also observed in the full replacement) but does not result in altered orientation of the maxillary palps nor heart-shape palps in males, while replacement of only the 3' portion of the locus results in a partial loss-of-function phenotype. These observations underscore the importance of replacing the entire locus to accommodate potential species-specific evolution of interactions among distant cis-regulatory elements.

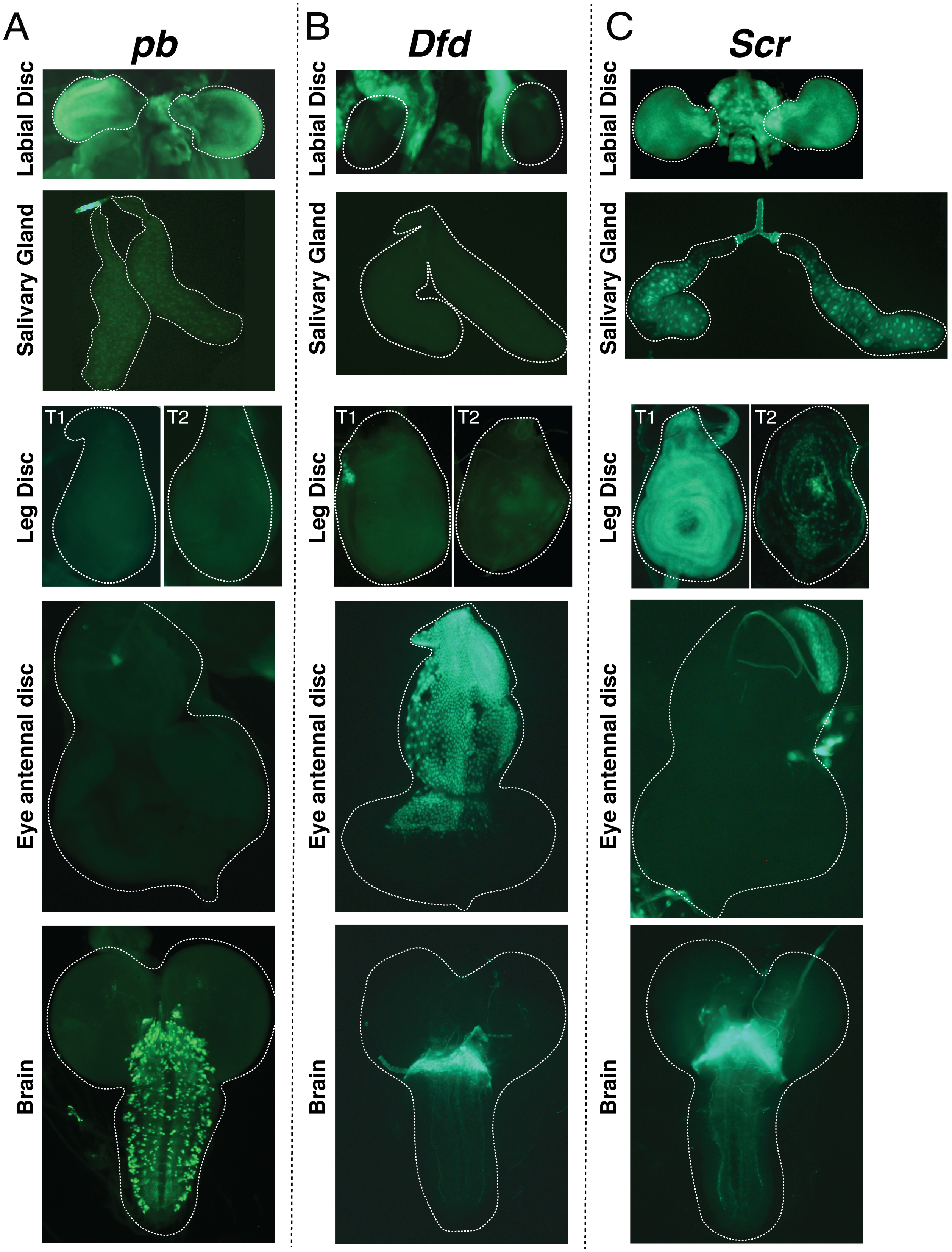

Fig. S1: Expression of the *pb, Dfd,* and *Scr* loci in third instar larvae. Expression of the *melPb-G4* **(A)**, *Dfd-GAL4* **(B)** and *Scr-Gal4* **(C)** genes was visualized by the *UAS-nlsGFP* reporter gene

(green) driven by T2A-GAL4 insertions into these loci respectively (Fig. 3A) in labial discs, salivary glands, leg disc T1, leg disc T2, eye-antennal discs and the brain/central nervous system.

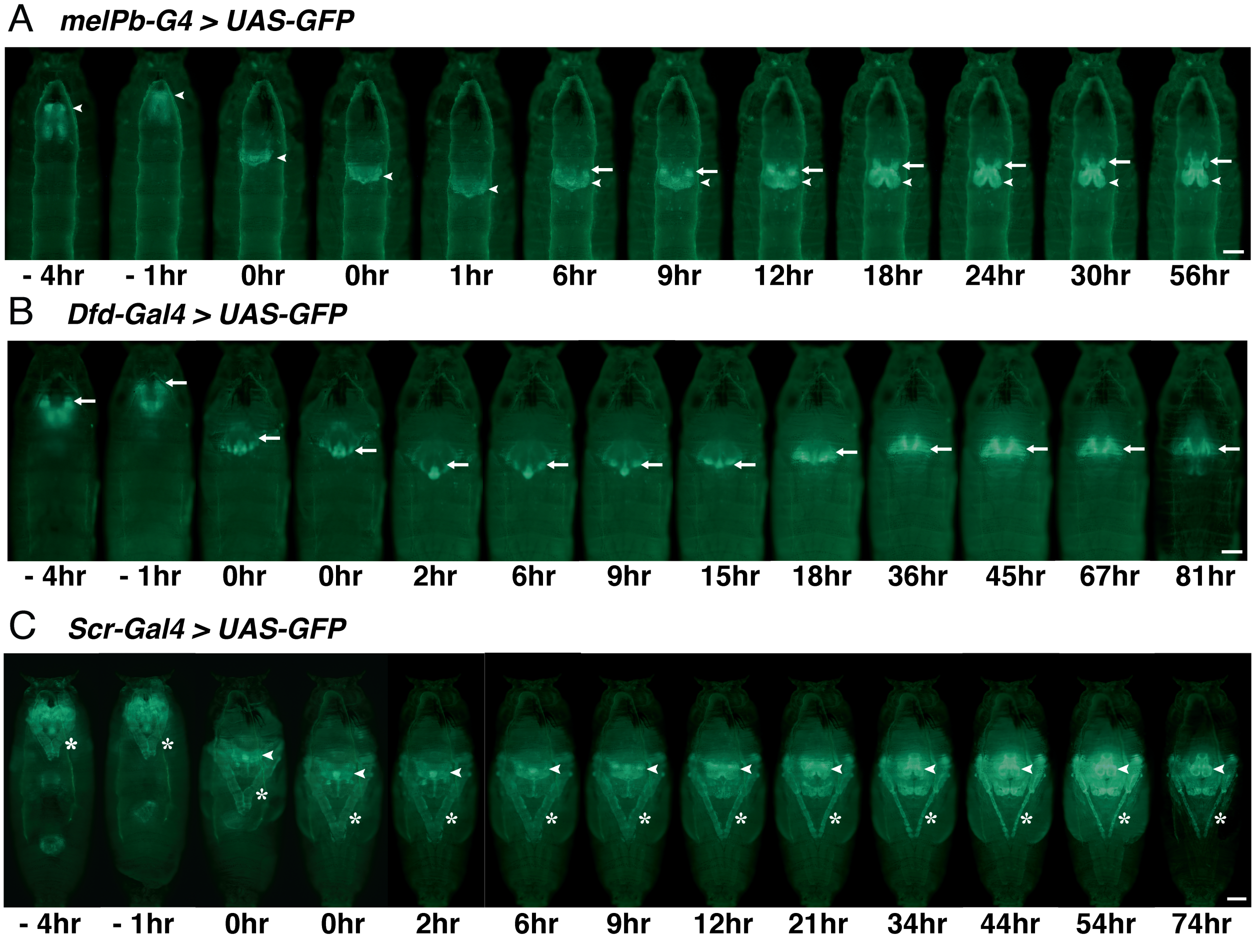

Fig. S2: Expression of *pb, Dfd,* and *Scr* during metamorphosis. The expression of *melPb-G4* **(A)**, *Dfd-GAL4* **(B)** and *Scr-Gal4* **(C)** in developing mouthparts using *UAS-nlsGFP* as the reporter gene (green). Each image represents individual frames from live imaging shown in Supplemental Data Movie 1, 2 and 3. The time the image was recorded is indicated (hr) and the arrowhead indicate the developing labial segment, the arrow indicates the maxillary primordia, and asterisk indicates T1 leg primordia expression. Scale bar, 250 µm.

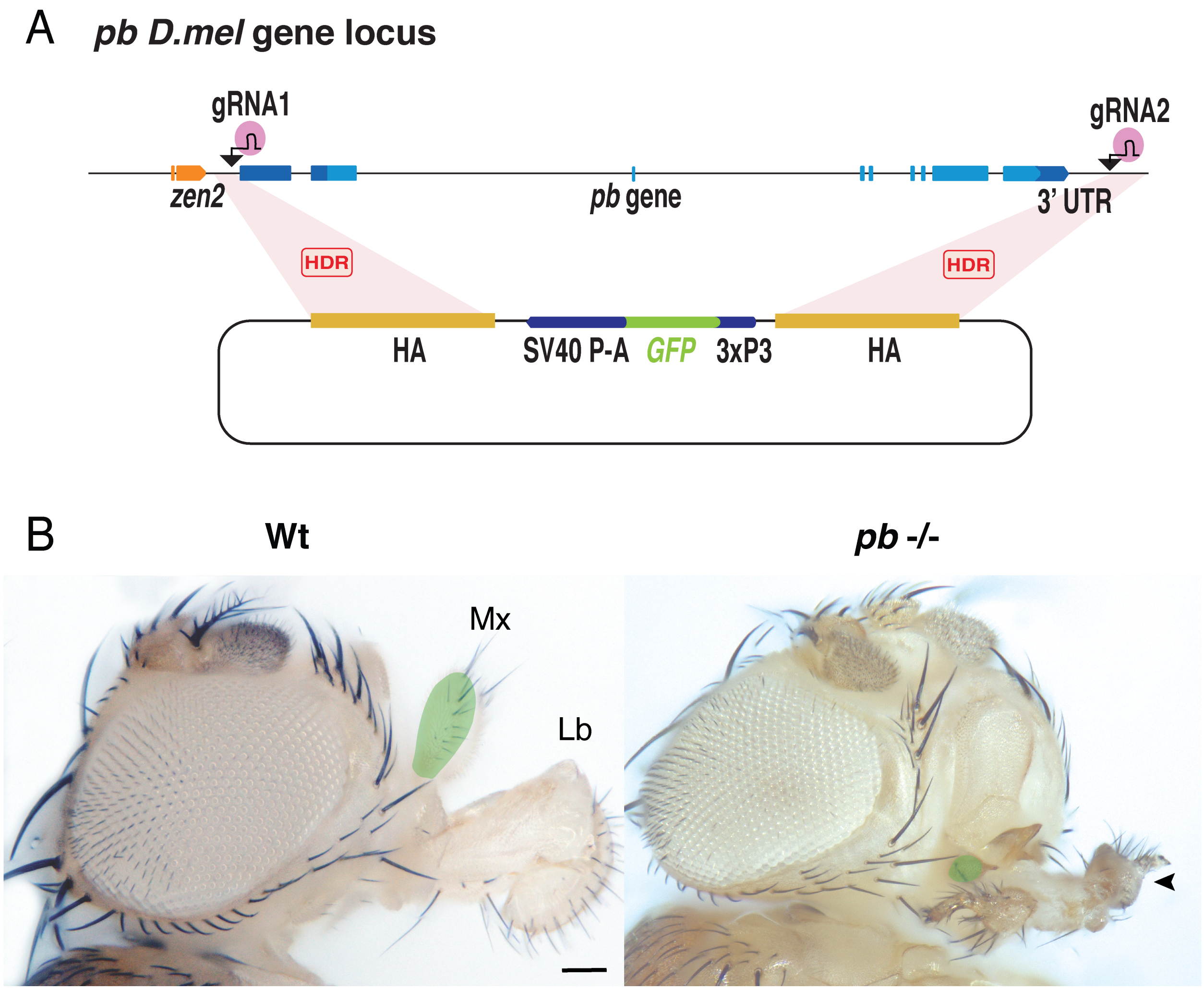

Fig. S3: Deletion of the *pb* gene locus in *D.mel* (A) Scheme depicting whole *pb* locus deletion in *D.mel pb*, with *pb* and *Zen2* gene introns and exons depicted on top. Also shown are the two gRNAs (gRNA1 and 2) and Cas9 used to delete the 38 kb *pb* locus and replace it with plasmid carrying an eGFP fluorescent eye marker cassette. **(B)** Fly heads from a *pb* wild type (Wt, *w1118* strain) and a *pb* gene locus deletion (*pb-/-*) fly head. False color indicates maxillary palps (green), and arrowheads indicate labellar lobes. Scale bar, 50 µm.

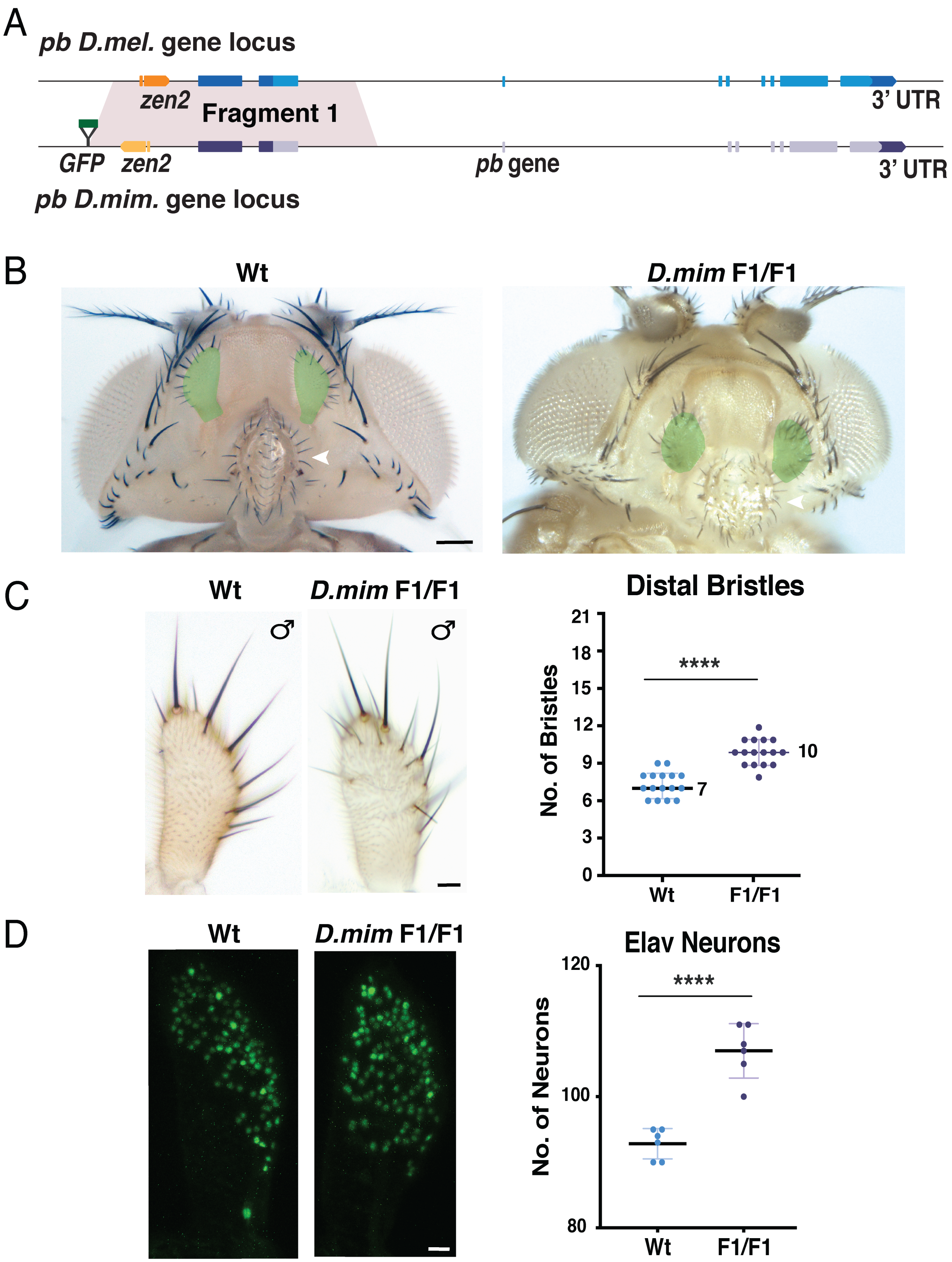
A
pb D.mel. gene locus
zen2
Fragment 1
3' UTR
GFP
zen2
pb gene
3' UTR
pb D.mim. gene locus
B
Wt
D.mim F1/F1
C
Wt
D.mim F1/F1
Distal Bristles
No. of Bristles
****
10
7
Wt
F1/F1
D
Wt
D.mim F1/F1
Elav Neurons
No. of Neurons
****
Wt
F1/F1

Fig. S4: Replacement of *pb* fragment 1 from *D.mim* to *D.mel* **(A)** Scheme depicting the entire *pb* genomic locus from *D.mel* (38 kb), along with the replacement DNA fragment 1 (*D.mim F1*) from *D.mim pb* with *3xP3-GFP* (GFP) marker cassette flanked by lox sites. **(B)** Bright field images of *D.mel* and *D.mim F1* replaced head. False color indicates maxillary palps (green), and arrowheads indicate labellar lobes. Scale bar, 50 µm. **(C)** Female and male maxillary palps of *D.mel* and *D.mim F1*replaced. Scale bar, 25 µm. Graph represents number of bristles on maxillary palp in *D.mel*, and *D.mim F1* replaced genotypes. Error bars indicate standard deviation; No. of maxillary palp scored (n)=16; ****p < 0.0001. **(D)** Female maxillary palp neurons *in the D.mel* and *D.mim F1* replaced animals, as visualized by staining with anti-Elav antibodies. Scale bar, 25 µm. Graph represents number of neurons in maxillary palp in *D.mel* and *D.mim F1* genotypes. Error bars indicate standard deviation; No. of maxillary palp scored (n)=6; ****p < 0.0001.

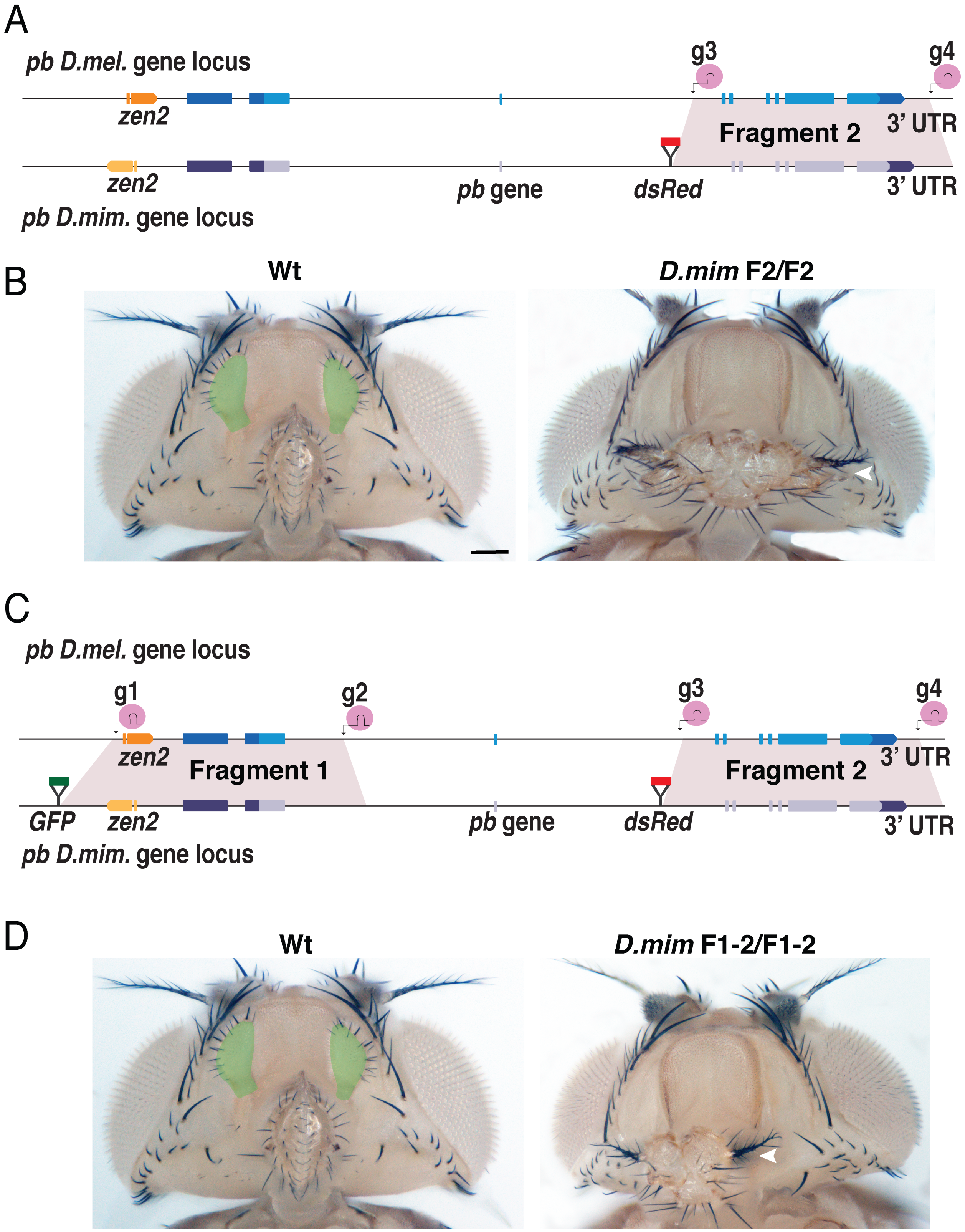

Fig. S5: Replacement of *pb* fragment 2 from *D.mim* to *D.mel* **(A)** Scheme depicting whole *pb* genomic locus from *D.mel* (38 kb) and a replacement of *D.mim* fragments 2 (F2) along with *3xP3-RFP* (RFP) marker cassette flanked by lox sites. **(B)** Bright field image of *D.mel* and *D.mim* F2

replaced heads. False color indicates maxillary palps (green), and arrowheads indicate labellar lobes. Scale bar, 50 µm. **(C)** Scheme depicting whole *pb* genomic locus from *D.mel* (38kb) and a replacement of both fragments-1 and 2 (*D.mim F1-F2*) replacement. **(D)** Bright field image of wild type *D.mel* and *D.mim F1-F2* replaced heads. False color indicates maxillary palps (green), and arrowheads indicate labellar lobes. Scale bar, 50 µm.

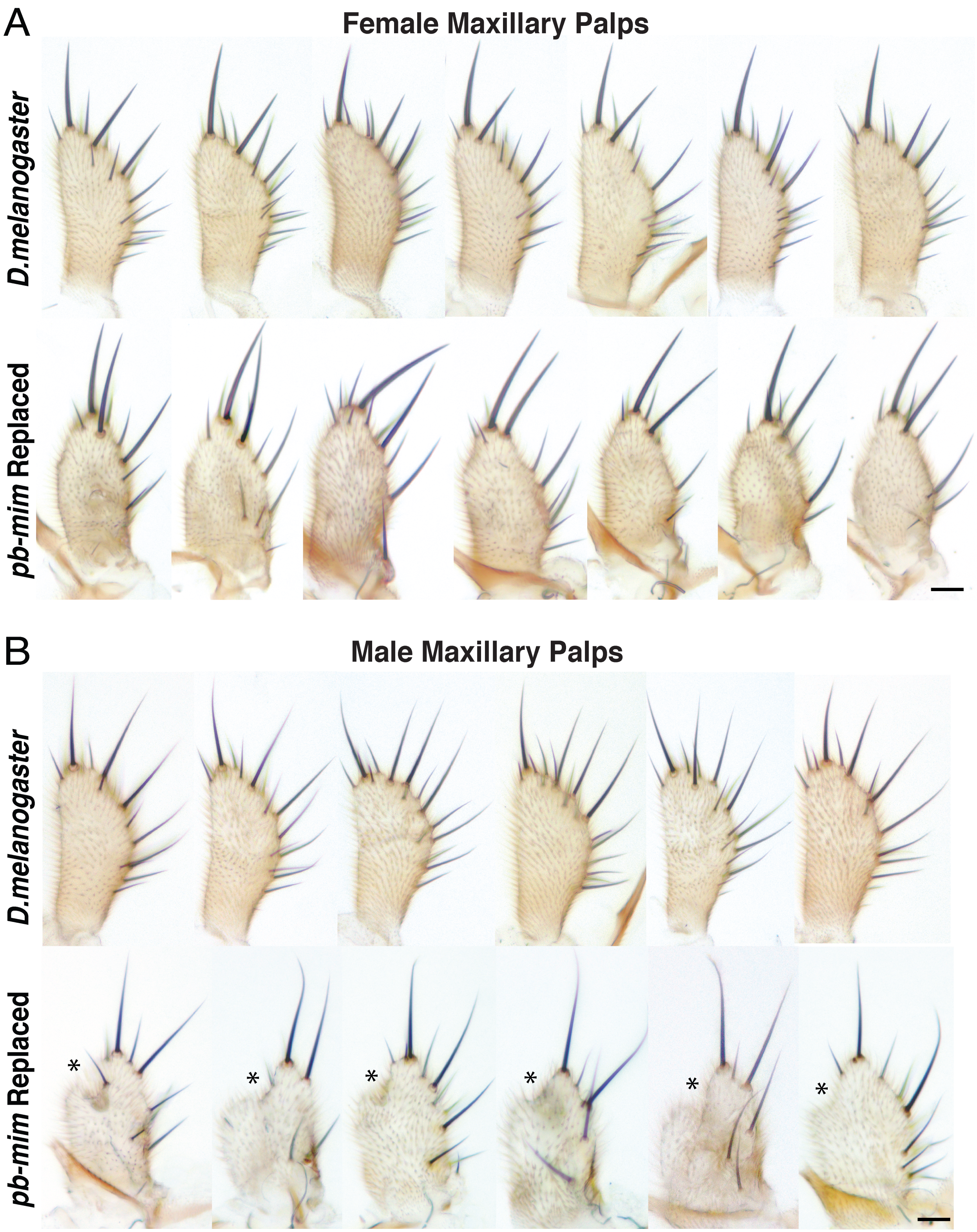

Fig. S6: Variation of maxillary palp phenotypes in *pb-mim* replacement. Array of maxillary palps from *D.mel* and *pb-mim* replaced females **(A)** and males **(B)**. Asterisks indicate notch on inner surface. Scale bar, 25 µm.

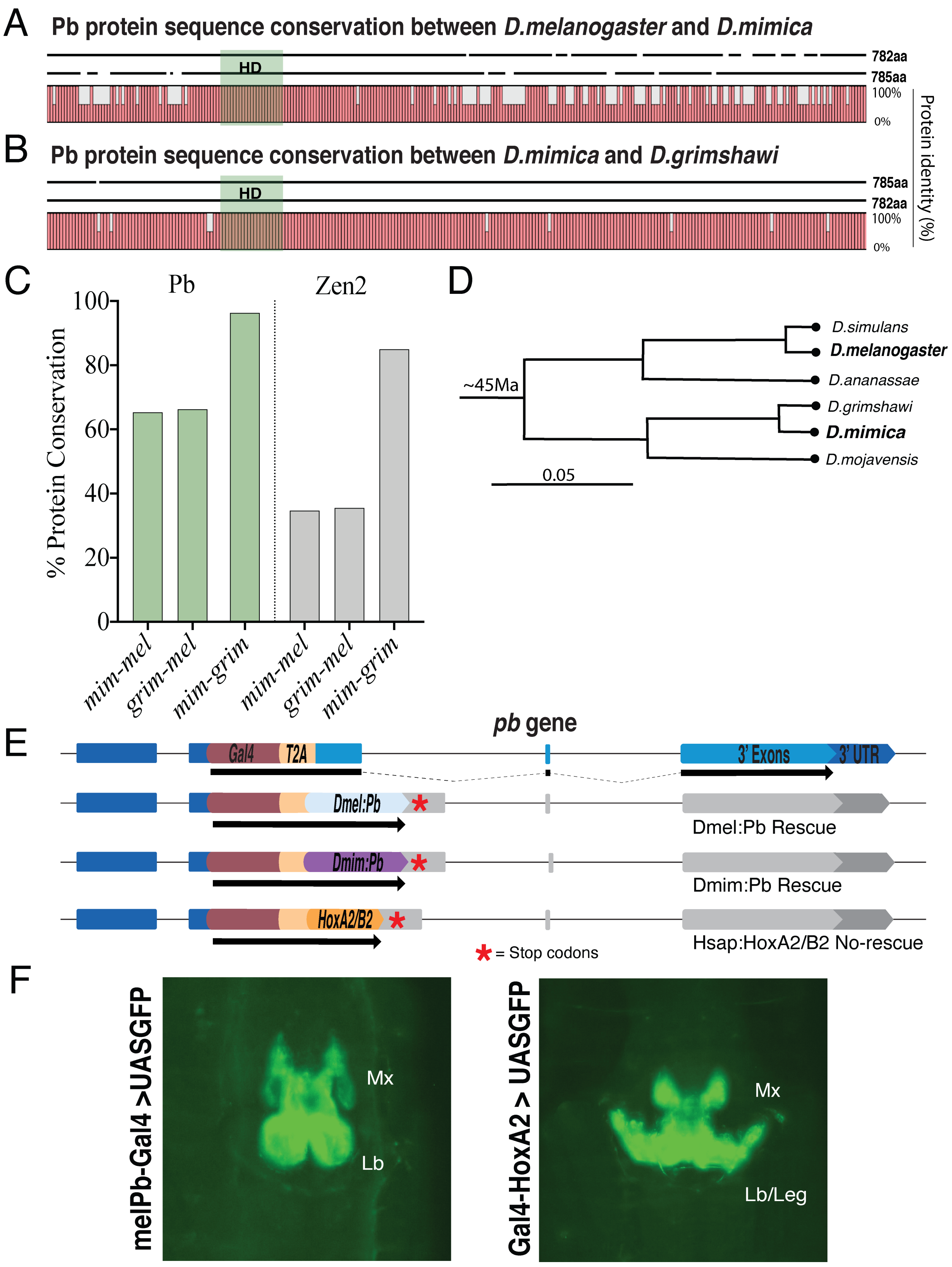

A
Pb protein sequence conservation between D.melanogaster and D.mimica
782aa
785aa
HD
100%
0%
B
Pb protein sequence conservation between D.mimica and D.grimshawi
785aa
782aa
HD
100%
0%
Protein identity (%)
C
Pb
Zen2
% Protein Conservation
100
80
60
40
20
0
mim-mel
grim-mel
mim-grim
mim-mel
grim-mel
mim-grim
D
D.simulans
D.melanogaster
D.ananassae
D.grimshawi
D.mimica
D.mojavensis
~45Ma
0.05
E
pb gene
Gal4
T2A
3' Exons
3' UTR
Dmel:Pb
Dmel:Pb Rescue
Dmim:Pb
Dmim:Pb Rescue
HoxA2/B2
Hsap:HoxA2/B2 No-rescue
= Stop codons
F
melPb-Gal4 >UASGFP
Mx
Lb
Gal4-HoxA2 > UASGFP
Mx
Lb/Leg

Fig. S7: Comparison of Pb protein coding sequences and function in *D.mel* versus *D.mim* (A) The Pb protein sequence alignment of *D.mim* compared to *D.mel* The bar graph represents the conservation percentage of amino acid sequences. **(B)** The Pb protein sequence alignment of *D.mim* compared to *D.grim.* The bar graph represents the conservation percentage between single amino acid. **(C)** Pb (green) *and* zen2 (gray) paralog protein sequence comparison plotting percentage of amino acid conserved between *D.mim* and *D.mel (mim-mel), D.grim.* and *D.mel (grim-mel),* and *D.mim* and *D.grim. (mim-grim)*. **(D)** Phylogenetic tree of Pb protein sequences of six *Drosophila* species. The tree was made using the maximum likelihood method with the CLC Main Workbench program. Bar indicates distance. **(E)** Schematic of the *pb* genomic regions engineered to incorporate Gal4-T2A fused to *HoxA2* or *HoxB2* cDNAs followed by 2 stop codons. **(F)** Images of control (*melPb-G4)* and *Gal4-T2A:*HoxA2 cDNAs driving expression of *UAS-nlsGFP* in primordia of the maxillary palps (Mx) and proboscis (Lb) in late pupae. The HoxA2 proboscis shape is altered because it has been partially transformed to legs.

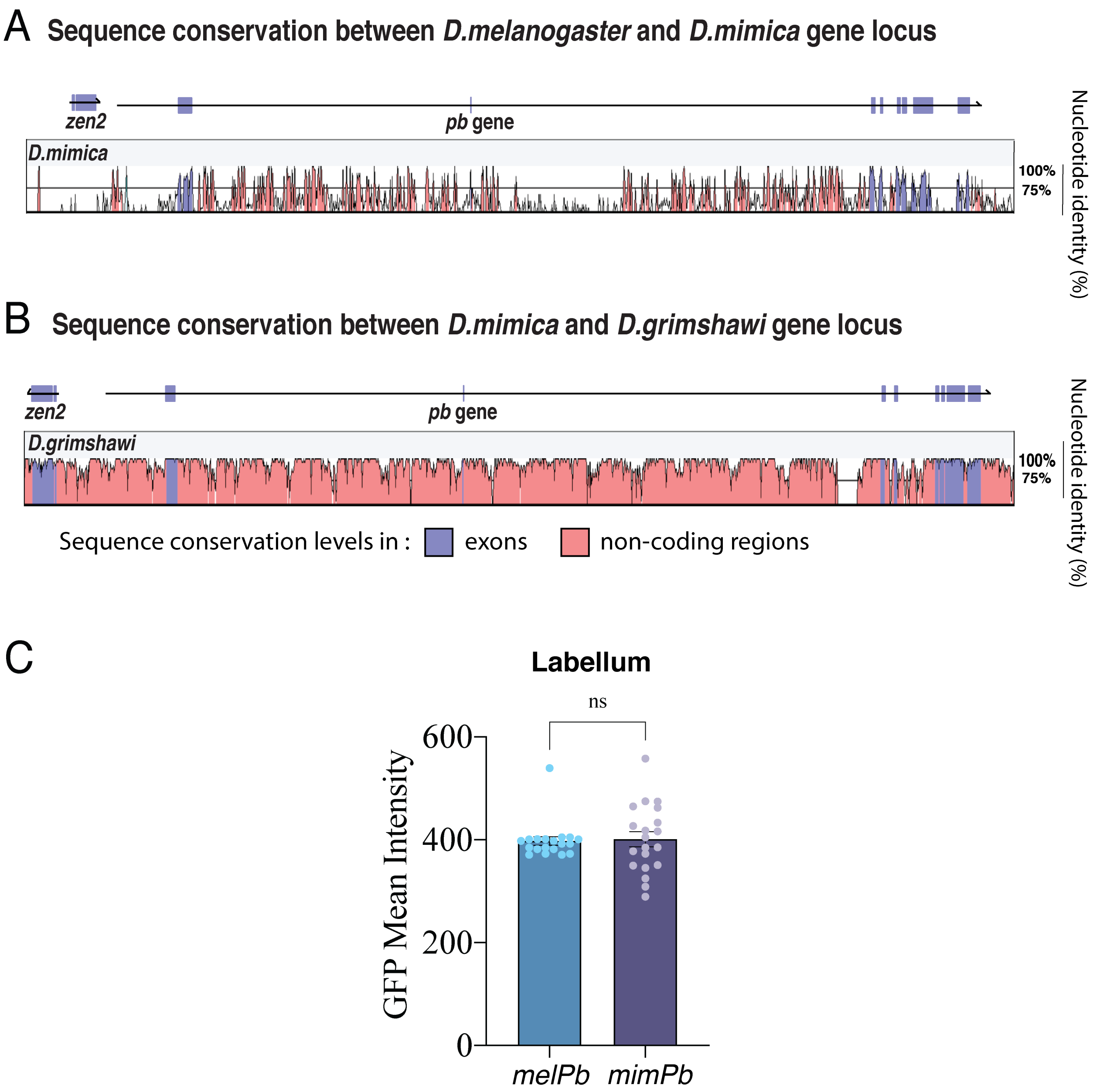

Fig. S8: Comparison of genomic *pb* sequences in *Drosophilids* (A) Genomic sequence conservation between *pb* loci in *D.mim* and *D.mel*. Blue regions indicate exons and red regions indicate conserved non-coding regions. **(B)** Genomic sequence conservation between *pb* loci in *D.mim* and *D.grim*. Blue regions indicate exons and red regions indicate conserved non-coding regions. **(C)** Approximately equal mean GFP intensities were measured in the labellum of late pupae for the *melPb-G4* and *mimPb-G4* reporters driving expression of *UAS-nlsGFP*. Error bars indicate standard deviation; No. of pupae scored (n)=20; ns >0.05.

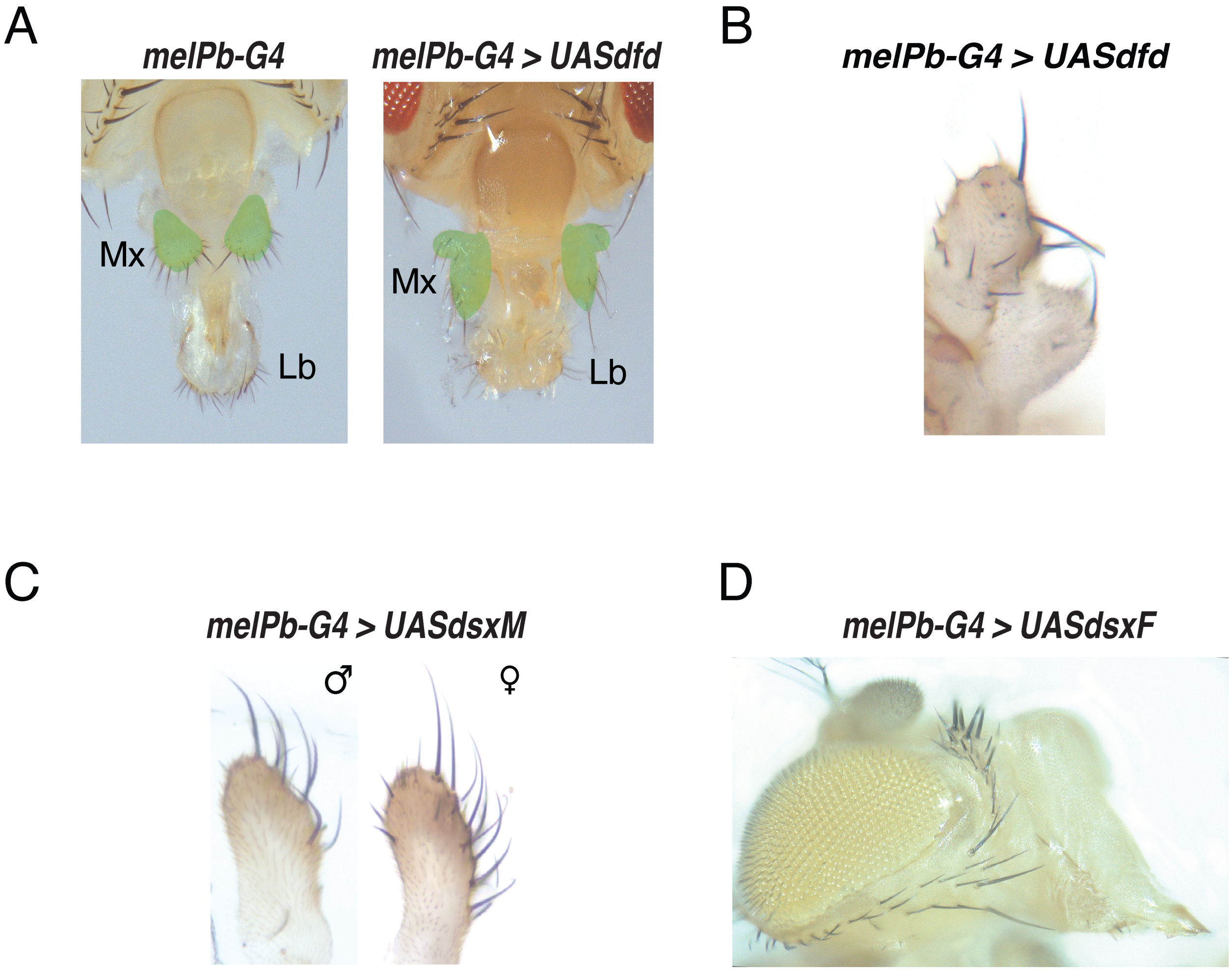

Fig. S9: Ectopic expression of *Dfd*, *dsxM* and *dsxF* in the *D.mel* pattern (A) Bright field image of *melPb-G4*, and *melPb-G4>UAS-Dfd* head. False color indicates maxillary palps (green) and arrowheads indicate labellar lobes. **(B)** Maxillary palp of *melPb-G4>UAS-Dfd*. **(C)** Representative maxillary palp of *melPb-G4>UAS-dsxM* male and female. **(D)** Bright field image of *melPb-G4>UAS-dsxF* fly head. Scale bar, 50 µm.

Table S1: Fly stocks

Source	Genotype
BDSC:4775	w[1118]; P{w[+mC]=UAS-GFP.nls}14
BDSC:7299	w[1118]; P{w[+mC]=UAS-Dfd.B}W4
BDSC:7302	w[1118]; P{w[+mC]=UAS-Scr.M}EE2/TM6B, Tb[+]
BDSC:44223	y[1] w[*]; P{w[+mC]=UAS-dsx.F}24-3
BDSC:44224	y[1] w[*]; P{w[+mC]=UAS-dsx.M}2/CyO
BDSC:7108	w[*]; P{w[+mC]=tubP-GAL80[ts]}10; TM2/TM6B, Tb[1]
In this study	w[1118]; melPb-Gal4
In this study	w[1118]; melPb-Gal4_T2A_cDNA:Pb
In this study	w[1118]; melPb-Gal4_T2A_cDNA:Pbmim
In this study	w[1118]; melPb-Gal4_T2A_cDNA:HoxA2
In this study	w[1118]; melPb-Gal4_T2A_cDNA:HoxB2
In this study	w[1118]; mimPb-gal4
In this study	w[1118]; Dfd-Gal4
In this study	w[1118]; Scr-Gal4
In this study	w[1118]; Pbdel
In this study	w[1118]; pb-mim Frag1
In this study	w[1118]; pb-mim Frag2
In this study	w[1118]; pb-mim Frag1-2
In this study	w[1118]; pb-mim Replaced

Table S2: gRNA sequences

gRNA	Sequences
Del1_gRNA	GCACCGCGCATGTGCCGACC
Del2_gRNA	GACGACTTGGGGTTGTATGA
F1a_gRNA	GGAAGGCAGTGCGATTAGCG
F1b_gRNA	GATCATCTGATAGGCTACTC
F2a_gRNA	GGGGTGGAGAGTGCTCCGGG
F2b_gRNA	GACGACTTGGGGTTGTATGA
F3a_gRNA	GGCAGTGCGATTAGCGTCTG
F3b_gRNA	GCGATCGTAGTGCCCCAACT
pbGal4_gRNA	GCAGACTTCTTGCATAGCTT
DfdGal4_gRNA	GCATGAGAAAAGAGCTCATGA
ScrGal4_gRNA	GTACCAGTTCGTCAACTCGC

Table S3: Primers *D. mimica*

**Oligos_mimica**	
**Fragment 1 Amplification Oligos**	
AA83	GTGGAAATCACTTTATGATGTGATGACACC
AA319	GGTTGAGACGGTTGCCGGGGGTGGAGTTCTGCC
AA320	GGCAGAACTCCACCCCCGGCAACCGTCTCAACC
AA281	CTACACATTCGCGCCACTAAAGTGCTGTG
AA282	CCAGAAAGGGACAGCTACACACAGCAC
AA237	GATTCCGTACGAGTGTCGGCGCTTGGAATGCTTCACGCTATTTATTGGC
AA238	CCAAGTCCGAGTGAAAGTGAGCCAATAAATAGCGTGAAGCATTCCAAGCG
AA239	GTATCTTGGCGCACTTATCTCCATAATAAAGTCGTAACAATTAATTGCGCG
AA240	GGCCTGAGCCACTGAAAAGGTCGCGCAATTAATTGTTACGACTTTATTATGGAG
AA352	CTGTGGCTGCCACTAATGTCGACAAC
**Fragment 2 Amplification Oligos**	
AA181	GAGAATAAGCATAATCCAGATCCACCCC
AA182R	CGAAACGAAACTTATCAGCAACTACGTC
AA246	GCTGCTGCACAAAGTATCCGACGTAGTTGCTGATAAGTTTCGTTTCG
AA247	CGGCGATTTTGAAACCAGACCTTAACCTGGAAGTGGTAGCAAAATGGTAG
AA186	CTACCATTTTGCTACCACTTCCAGGTTA
AA187	CCAAAGTTAAAATTTTCCAGGAGAGCCC
AA217	GAGACAGACATCTAGTTTAGGGCTCTCCTG
AA218	CAGCCCGAGTTGCCTGATTTTGTTGTAAC
**Fragment 3 Amplification Oligos**	
AA240	GGCCTGAGCCACTGAAAAGGTCGCGCAATTAATTGTTACGACTTTATTATGGAG
AA241	CAGTAGGTGTGACCAGTCTACTCATTCATTCTGACAGTTTTCCCACCCAC
AA120	GAGTGGGTGGGAAAACTGTCAGAATGAATG
AA178	GTCGGCTACGCACTATTGTAATGAAAAC
AA191	CACTCCGACACTCTGACTCTTATGGCCG
AA192	TCCTCAATAGCCGGACTTCTGTTTCCGC
AA242	TAGGCTAGACAAAGCTTACTTAAAGCGGAAACAGAAGTCCGGCTATTGAG
AA243	ATGGCCGACTCTCTCTGGCTGGGGTGGATCTGGATTATGCTTATTCTC
AA244	CGCTTTCAATCTGGCAAACATGCTGAGAATAAGCATAATCCAGATCCAC

Table S4: Primers

Sequencing oligos	
AA104	CAAATGTGTCAGAGCTGAAACGTCATGATTAATG
AA106	CATCATTAATCATGACGTTTCAGCTCTGACAC
AA107	TCGTCGGGGCAGGCCGTTTTCTG
AA107	GGAAATGTCATCAGCATGGATGAAATGCCG
AA108	GCAGCTGAATTCGTTCCAGAAAACGGCCTGC
AA119	CATTCATTCTGACAGTTTTCCCACCCACTC
AA121	CCATCAGGGATGCTAAGAGCTGTCAATC
AA121	CCATCAGGGATGCTAAGAGCTGTCAATC
AA122	GATTGACAGCTCTTAGCATCCCTGATGG
AA122	GATTGACAGCTCTTAGCATCCCTGATGG
AA123	CGAATTGGAGTCTGCATTTCACGGG
AA124	CCCGTGAAATGCAGACTCCAATTCG
AA146	CACGCACATCGAACAAAATCAG
AA147	CGCACTTGGTCGCGGTTTGTTC
AA147	CGCACTTGGTCGCGGTTTGTTC
AA148	CATGATACTTGCGTGGTACG
AA148	CATGATACTTGCGTGGTACG
AA149	CCTCATTGGGTTCAGGACAC
AA150	GCAACTGCGAGTCAAGTGCC
AA151	GGCTGCATTTGTTCTGCGAATTG
AA152	GCCGTGGAAGTTGAACTCG
AA152	GCCGTGGAAGTTGAACTCG
AA153	CAACGGGAGTGGTAACTTCAACTCC
AA154	GGAGTTGAAGTTACCACTCCCGTTG
AA155	GGATGGATACAGGGAGCGGG
AA156	GTGTGCCCCGCTTGCTACGC
AA157	GCCAGGCATCACATGCAATCG
AA158	CTTGGTGCAGGGCGCGCTG
AA159	GGGATCTCCGGACTCAAGTG
AA160	CGACTCGGATGTTGGTGCTG
AA160	CGACTCGGATGTTGGTGCTG
AA161	CGTCTGCAAATGGAAGTCAATGC
AA176	CGAGTTGTGGAGATCTACCG
AA177	CGCGCCACTAAAGTGCTGTG
AA180	GGGGTGGATCTGGATTATGCTTATTCTC
AA180	GGGGTGGATCTGGATTATGCTTATTCTC
AA181	GAGAATAAGCATAATCCAGATCCACCCC

AA184	GACGTAGTTGCTGATAAGTTTCGTTTCG
AA185	TAACCTGGAAGTGGTAGCAAAATGGTAG
AA189	GCATGGGCTTCGTGTAAGATCGCAACAA
AA190	TGCAAATCTTCAGTTCAGGCAACCAGGC
AA193	GCGGAAACAGAAGTCCGGCTATTGAG
AA194	TAAGCCTCTGGTCCATAGACCTCTCCCC
AA194	TAAGCCTCTGGTCCATAGACCTCTCCCC
AA197	AAGACTCGTATACCCTGTGG
AA198	GCAGCTGTTGGCCGTTGCTG
AA199	GCACAGCAGCCAATTCAACA
AA200	CACATGCACTTGACATGGCC
AA201	ATTGATGATGCGGCTTTGGC
AA202	CCCATAAGCTGCCTAATGGC
AA202	CCCATAAGCTGCCTAATGGC
AA203	GGAGGGTCCTCCGAACAGAAG
AA203	GGAGGGTCCTCCGAACAGAAG
AA204	CATCTCTGCGCATTCGTACG
AA205	GCGAGCTTTCGTTGTCCAGC
AA206	CAGTGTCTATGTCTGGCTGC
AA208	CGGATGATCAACGTCCAACG
AA209	CCCGTCTACTCACGCGACTG
AA209	CCCGTCTACTCACGCGACTG
AA211	CGGCTCTTGGACTCGGTCTG
AA212	GCCAAGTTGGAAGGCATTGG
AA213	CACTTTGTGCGCACTGGCAG
AA214	GCTGCCACGGTCACAGGACT
AA215	GCTTGCGGCTGCTCACAGCA
AA216	GCCTCTTCATCTCTGGCTGG
AA216	GCCTCTTCATCTCTGGCTGG
AA230	GTACACACTTAGTAAGTGCCACTTAAAACTCTACGCTTGC
AA231	CTGTGGCTGCCACTAATGTCGACAACTTTAGT
AA264	CATGGCACTTGACTCGCAGTTGC
AA264	CAGTTTGCAGATAAGGACTGACCGC
AA276	ACTCGGTGCCCGAGTATCCCTGGATG
AA281	GCGCAGCGTTTGCTCAAAATAATTGC
AA323	AGACCGAGCTGTCCATCAAAGGAGCCGAAC
AA348	GCAGCTCGACATGATGGAAATGGAACTGTG
AA434	TGGGTTCCAGCAACAGCACC

Table S5: Maxillary palp Normalized GFP intensity

Pbmel_F	Pbmim_F	Pbmel_M	Pbmim_M
46.19951	51.31782	59.16485	48.7396
42.48317	44.69703	67.46287	52.63787
69.6953	60.86609	58.07277	38.97624
67.52748	46.04604	44.36089	33.70371
63.08936	53.90941	58.88911	32.82252
77.16411	55	54.98317	42.98094
66.83144	36.79876	45.97104	41.58614
65.5349	34.76807	54.54604	43.39059
51.55545	58.85272	59.16485	46.3896
54.95545	30.77401	65.46287	40.86262
44.06436	40.80743	58.07277	38.77327
51.66609	36.10767	44.36089	42.00965
60.19233	79.36386	58.88911	28.69183
68.6255	61.24233	54.98317	38.41287
50.40866	45.79802	45.97104	54.75718
42.56436	53.11238	54.54604	31.35272
43.57302	46.04134	47.55495	35.81188
48.44505	60.31757	49.48812	38.11634
52.53342	51.31782	55.81436	34.64653
68.99381	44.69703	42.56436	32.52401
47.55495	60.86609	43.57302	37.31114
49.48812	46.04604	48.44505	32.71708
55.81436	53.90941		
62.02946	54.98317		
58.61906	36.79876		
57.33861	34.76807		
49.64183	58.85272		
56.0255	40.80743		
53.32129	36.10767		
54.34158	79.36386		

Table S6: Maxillary palp raw GFP intensity

Pbmel_F	Pbmim_F	Pbmel_M	Pbmim_M
186.646	207.324	239.026	196.908
171.632	180.576	272.55	212.657
281.569	245.899	234.614	157.464
272.811	186.026	179.218	136.163
254.881	217.794	237.912	132.603
311.743	222.2	222.132	173.643
269.999	148.667	185.723	168.008
264.761	140.463	220.366	175.298
208.284	237.765	239.026	187.414
222.02	124.327	272.55	165.085
178.02	164.862	234.614	156.644
208.731	145.875	179.218	169.719
243.177	320.63	237.912	115.915
277.247	247.419	222.132	155.188
203.651	185.024	185.723	221.219
171.96	214.574	220.366	126.665
176.035	186.007	192.122	144.68
195.718	243.683	199.932	153.99
212.235	207.324	225.49	139.972
278.735	180.576	171.96	131.397
192.122	245.899	176.035	150.737
199.932	186.026	195.718	132.177
225.49	217.794		
250.599	222.2		
236.821	148.667		
231.648	140.463		
200.553	237.765		
226.343	124.327		
252.978	164.862		
215.418	145.875		
250.038	320.63		

Table S7: Labellum GFP intensity

Pbmel	Pbmim
397.567	433.361
401.159	384.79
392.197	474.586
381.725	289.157
404.983	385.276
372.767	426.509
370.844	372.861
400.584	474.345
384.702	345.47
539.477	324.968
397.567	377.983
401.159	417.626
392.197	416.139
381.725	404.574
404.983	308.507
372.767	349.938
370.844	350.712
400.584	464.522
384.702	557.656
	462.451

Movie S1: The expression of melPb-GAL4 during metamorphosis.

Movie S2: The expression of Dfd-GAL4 during metamorphosis.

Movie S3: The expression of Scr-GAL4 during metamorphosis.

Data S1: *D.mim* zen2 and Pb protein and DNA sequences
