# Supplementary material for: Dissecting the evolutionary role of the Hox gene proboscipedia in Drosophila mouthpart diversification by full locus replacement
Source: Sci Adv. 2021 Nov 10;7(46):eabk1003. doi: 10.1126/sciadv.abk1003 (PMC8580299; doi:10.1126/sciadv.abk1003)
Supplement: Supplementary file 3 — Data S1 [file sciadv.abk1003_data_s1.zip › sciadv.abk1003_data_s1.docx]

>Zen2_mimica_protein

MYNNCSPSESFTANASNYMLNCEVQPSGFPCPPSTTKCKRSRTAFTSHQLLELEREFNENKYLGRPRRIGISRRLLLTERQVKIWFQNRRMKSKKLANRQLGHKGSKLLLDCGIVQGEEYLNQPQLSEDEMIVERLLQYVSTGQDSLTCDLSDPRNDYAIMEQRQTSFNAGKCPEPNEDRLPSASQEYSGVDTWPTSWFNTEQWVATSDEQELQNINHFTADPFVNPQMAWESSYSVGSSASTSTASCDSFDIQEFQNIDSFYVDQSLDKQFGWDSSGSIAVSTTNSTASCDSFEPLDVDYDFLQHLLDA*

>Pb_mimica_Protein

MQEVCSTLDTTQMGAQIKSESPLNPLQVQTGQTTVVPVVTGPGPQGPPPAVMLVNKMAPNCDKRAADTAYWMASEGGFINSQPSMAEFLNHLSPESPKIGTPVVGGGVGGGYPVGVGGVPQTPDGMDSVPEYPWMKEKKTSRKSSNNNNQGDNSITEFVPENGLPRRLRTAYTNTQLLELEKEFHFNKYLCRPRRIEIAASLDLTERQWFQNRRMKHKRQTLSKTDDEDNKDSLKGDDDQSDSNSNSKKSCQGCELPSDDIPDSTSNSRGHNNNTPSATNNNPSAGSLTPNSSLETGISSNLLGSTTVSASNVISADSSVASSVSLDEDIDESPIKVKKKDDTHGQVIKKEAVSTSSKASPFGYTDAGPSLASFRRDSDASVASNPPVSKAGNKKRYQNPNANANPIGIASPLSESSNAAGPAGYFPGAGYYPNPNVNPGAKALQAPQQMPQDYYGKYDIEFAASPHHNPHKQQQQQQQPLHGEYLSPKPNTNAANSNFHQNSQQQQHQHEQQFYYNYNDTNGGSAYMNHQQHQQQHHPVGDFEAPPINGPTNFYDPKSQTSGAYYDNMNFQHQHQSVGFQQQHQQQHQQQQTPINHQQHMHHIGAGETYSALGLQMENCENYNNFGGGYYEPGAAQQQQQQPPGPPTHTHPHPHPHPHPHHPHPHPHHMQAQAHPHLHASHHNPVATAATVQVVGGGAPPPPTSHVHIPNANAANSNFVMNGGGAPVVTGGQIQAFANTGGGGGVAAAAAISGLENSNSSSDFNFLSNLANDFAPEYYQLS*

>pb_cDNA

ATGCAAGAGGTGTGCAGTACATTGGACACAACCCAAATGGGCGCCCAGATCAAATCGGAGTCGCCACTCAATCCGCTTCAGGTGCAAACGGGACAGACGACGGTGGTGCCCGTTGTGACCGGACCGGGGCCGCAGGGACCGCCGCCAGCGGTGATGCTTGTCAACAAAATGGCACCGAACTGTGATAAACGAGCGGCGGACACCGCCTATTGGATGGCCTCCGAGGGGGGTTTCATCAACTCTCAGCCCTCGATGGCCGAGTTTCTCAATCACTTGAGTCCGGAGAGCCCGAAAATTGGCACACCTGTTGTCGGTGGCGGTGTCGGTGGCGGATATCCGGTCGGTGTTGGTGGTGTACCGCAGACACCGGATGGGATGGACTCGGTGCCCGAGTATCCCTGGATGAAAGAGAAGAAGACATCGCGCAAGAGCAGCAACAACAACAATCAGAGGTGATAACTCCATAACTGAATTCGTTCCAGAAAACGGCCTGCCCCGACGACTGCGCACCGCGTACACAAACACGCAGCTGCTGGAGCTGGAGAAGGAATTCCATTTCAATAAATATTTATGCCGCCCAAGGAGAATTGAAATAGCAGCCAGCTTGGATCTGACCGAGCGGCAGTGGTTTCAAAATCGCCGCATGAAACACAAGCGACAAACGCTCTCCAAGACAGACGATGAGGACAACAAGGACAGCCTCAAAGGTGACGACGATCAATCCGACAGCAACTCCAATTCGAAGAAATCGTGTCAAGGCTGCGAGCTGCCCTCCGATGATATACCGGACTCCACGTCCAACTCGAGAGGACACAATAACAACACGCCCAGCGCCACAAATAACAATCCGAGCGCAGGAAGTCTCACTCCGAACTCATCACTGGAGACTGGTATCTCGTCCAATCTGCTGGGCAGCACCACCGTATCCGCCTCGAATGTCATCAGTGCCGACTCTAGCGTCGCGTCCAGTGTCAGCCTCGACGAGGACATCGACGAGAGTCCCATCAAGGTCAAGAAGAAAGACGACACTCACGGCCAGGTGATTAAAAAGGAGGCTGTTTCCACCTCGTCCAAGGCCTCACCCTTCGGCTACACCGACGCGGGCCCCAGTTTGGCCAGTTTCCGGCGCGATTCCGATGCCTCTGTTGCCTCGAATCCGCCCGTATCCAAGGCCGGCAACAAGAAACGGTATCAGAATCCAAACGCGAATGCTAATCCAATTGGAATTGCGTCGCCACTGAGCGAGAGCAGCAACGCTGCTGGGCCAGCTGGTTATTTTCCTGGTGCTGGCTACTATCCAAATCCGAATGTCAATCCGGGTGCAAAAGCATTGCAGGCGCCGCAGCAAATGCCGCAGGATTATTATGGCAAATACGATATTGAGTTTGCAGCCTCGCCACACCACAACCCGCACAAGCAACAGCAGCAGCAGCAACAACCGCTTCACGGCGAATATCTAAGTCCCAAACCAAACACCAATGCCGCCAACAGTAATTTCCATCAAAACAGTCAACAGCAACAACATCAACATGAACAGCAGTTCTACTACAACTACAACGACACCAATGGCGGTAGTGCGTACATGAACCACCAACAGCACCAGCAGCAGCATCATCCAGTTGGTGACTTTGAGGCGCCACCTATCAACGGACCGACGAACTTTTATGATCCCAAGTCGCAAACCAGCGGCGCTTACTACGACAACATGAATTTCCAACACCAACACCAGTCGGTTGGGTTCCAGCAACAGCACCAACAACAACACCAGCAACAGCAGACGCCAATAAATCACCAACAGCACATGCATCACATTGGAGCCGGTGAAACGTACAGTGCGCTCGGTCTGCAAATGGAGAACTGCGAGAACTACAACAACTTTGGCGGCGGCTACTATGAACCGGGCGCTGCTCAGCAACAGCAACAACAACCGCCTGGGCCGCCCACTCACACCCATCCCCACCCTCATCCGCATCCACATCCCCATCATCCGCATCCGCATCCTCATCATATGCAGGCGCAGGCGCATCCACATCTACATGCATCGCACCACAATCCAGTTGCGACTGCAGCCACTGTCCAAGTGGTGGGCGGAGGAGCGCCCCCGCCACCCACCTCTCATGTCCACATACCAAATGCAAATGCGGCGAATTCCAATTTCGTGATGAACGGGGGTGGTGCGCCTGTGGTGACTGGTGGACAGATACAGGCGTTTGCCAACACTGGTGGTGGTGGTGGTGTTGCGGCAGCGGCTGCCATCAGCGGGCTGGAGAATTCGAACAGCTCGTCGGACTTTAATTTTCTGAGCAATCTGGCCAACGATTTTGCGCCCGAATATTATCAGCTAAGTTAG

>pbmim_replaced_locus

TGTATAAGCTCATGAGTGTCCTGTGAGTGCCTGTGTGTGTGTGTGTATGTGTGTGTGTGTGTGTGTAAGTATCCGTTTTCTTTGTTGAATGACCAAAATTTCAATATGTGGAAATCACTTTATGATGTGATGACACCAATTTATCTAAGTTAGTTGCCAACAGCCGGCAAGTGGCAGATACGCGTAAAGCCACTGAATACTCGAATGTGTAGCAGTTGCCTCCAAGATGGGGCAGATCTTTTATCTTGCTTTTGAAATTTATTAGAAAATACAAATTGCTTGTTTTAATAAATTACATTTCTATGAAATATTTATGTTAATCGTAGCTTAAGTTTAATTTAATTACATAAATTATATTAAACAATAAATTACTTGCTTTTATGCATCTAATAAATGTTGCAAGAAGTCATAATCAACATCAAGGGGTTCAAAGGAATCACATGAAGCTGTCGAATTGGTTGTAGACACTGCAATCGAGCCACTCGAATCCCAGCCAAATTGTTTATCCAAAGATTGATCCACATAAAACGAATCGATGTTTTGAAACTCTTGAATGTCAAATGAATCACACGATGCAGTAGATGTGGATGCAGATGATCCCACCGAGTAGCTTGATTCCCAAGCCATCTGTGGATTCACAAATGGGTCAGCAGTAAAGTGATTGATGTTTTGCAATTCTTGTTCATCACTTGTGGCCACCCATTGCTCTGTGTTAAACCAACTGGTTGGCCAAGTGTCCACACCTGAATACTCTTGACTCGCACTTGGAAGCCGATCCTCATTGGGTTCAGGACACTTTCCTGCATTGAAGGAGGTCTGTCTCTGTTCCATAATAGCATAATCATTTCGCGGATCTGATAGATCACAAGTCAAGCTATCTTGGCCGGTGCTAACGTATTGCAAGAGACGTTCAACAATCATCTCATCTTCACTAAGTTGTGGTTGGTTTAAGTATTCTTCACCTTGCACGATGCCACAATCAAGCAACAGTTTGGAGCCCTTGTGTCCCAGCTGCCTATTGGCTAGTTTCTTGGATTTCATGCGTCGATTCTGGAACCAAATCTTAACTTGTCTTTCAGTCAACAGCAGACGCCGAGAGATTCCAATGCGACGCGGCCTTCCCAGATACTTATTCTCATTGAACTCCCGCTCCAACTCCAGCAGCTGATGGCTCGTGAATGCGGTGCGGGAACGCTTGCACTTGGTGGTGCTGGGTGGGCAGGGGAACCCAGAAGGCTGAACCTCACAGTTCAGCTAGTAAGAGTAATTGGTAAACATATATTGTAGAAGCTATTTAGCTGATATTTAGCTGTATGCAAACTTACCATATAGTTGCTTGCATTGGCCGTAAACGATTCACTGGGACTGCAGTTGTTGTACATTTTGTCGAGAGATATTTTAGCTTCTGCACTGTGACACATTGGTTTTTTAACTAGATGCGAATCCATGCTGTCGATTTACTTTTATACCATGCAGGTAAGCCGCTTGGCCGCGAATTTGGAGAACTCTTCCCAGTTGCTACCTGAGACCTGTGATCGGCGTTAGGCCTGACCAGCCGGCTGGTGCAACGTGTCTAGTCTGTTGGCGTGTCCAGGCTGTGGCTCCGGAAAGTCCGCAACACATTTAGAACGTGGCGTGAGTCTTGATTGTGAAACGACTGTGTCGTGTGTGTCTCATTGAAATTCACAATTTAATTGACTTTGCCAGGCATCACATGCAATCGAAAGTCCGTAACGATTTTATATTTAATTGTCCAAATAAATCTTAGAAGCCTTACATTTACTCAATATTGCCCGAACGCACTCAATATTTAATAACCCTAGTAAGACTTTTACTTTCATACCTGAATTTTAAGCAAGATAGTACTTTAGGTAAATCCATACTCGGCACCGAGATATGAAAGAAAATTTTCGTGGTTTTCCCCGGGGTTTTCCAAAATCGCTCTGACTACTTCACCTTGAGCTACCTGCACATTTTGGCTTAGGGTGACGAAGACCTCGAATGGGGTCATAAAGCACCCTGCATCGGTTAAACTTGATCTCCCTCAATAAAACTAGAGCTGATATTCTGCTTATATGTTTCCACCTACGAGTTTTATGATGAGGAGCTAAGGAAACTCGTACAGGCTTTTGCAAAATAACCTCAAATCTGGGCTTCAAAGTGTAAAGTATTTATGCATATTTACTTATGTAGGTAGGTGTTCTTATATATATGAACATATATAACTTATACCTCACACTCTCGTTTCTTTTTGTATTTCCAAATTAAAGTGTAAAGTTTTTACTTGAATTCGATCCTGATCATCGTCCAATTTAAGAATATATTCATGTTTCTATAGCTTATGGCATCACAGACGTCAATAATACCCTCTGCTAAGGTTTTTAAATACAAACAAGTTAGAACGCAATAGTCGTGACAGCTCGACTAGTAGATCGAAGACTTAATCGGATTTGTACAGCTCATATTTCAGCTGCATCTGTGTAATTATACCTTTTATTCGGATATATAATTGAAACGATGAAAGTTATCGATAAAATTAAAATAATGCTTGTACATACAGATGCACAAAGGGACACGGCTAAATCGACTCGGATGTTGGTGCTGATCAGGAATATGTATACTATATCTACCTATTACATACTCTTACACAACTGCATTATATAATTTTTCGAGTCTTAATTCCAGATATAAAAAGATGTTGACACTTTTGCATGTGTATTCTAACAAGTATTAGCAAATCGTACACTAAACAGAGCTTAATTGTGCATACTACATGTCTGGTGGTTCATAAACTTTCGGCTTTCACTGTGCTGCGAAAATGAAACAAGCGGCAGCGATGCGATTGCCAATAAAAACAGAATGTGAAAAAGTGTGGCAGCAGAAAAACAATGCCACCAAGATTGACAACATGATGGTTAGACAAGAAGAGGCGCTCAAAACAAAAAGAAAGACGCGCAGCTCGCAAAACGAAATGTCAAAAATACGCAACGCAAAAGCGACAGCGGAGACGCCAGCAAAAAAAGAAATGAAACTCGTCGCTGCCGCTTTTACTGCTGCTGTTGCTGCTGCTGCTGCTGCTGCTGCCGCTGCCACAGCGGCGACGGAATGTGTAAATAACAAAAAGCAAATAAATTCCGTCACAAGCAACACGTTCACAGTGCAGAGTGGTAAAAACAGCGGCGCAGCTGAGCGGACAGCCAAAGCGACACAGCCACAAAGCGACAGAGCGACAAAAACACACAACACAAGCAGCAGCAGCAATCGACCAATCAGCGCCAGCGGATACTTGTAGTGTGTCTGCATGTGTGAGTGAGTGCGAATCACTTGCATGTGTGTGTGCGTGCGTGTGAGTGTTTCTTACCTGTGTGCGACTGGCAGAACTCCACCCCCGGCAACCGTCTCAACCATCGCTATTGCGCATGTACAGCTCTGCTCTCTGCTGCCTGTGCACGAGTTGTGGAGATCTACCGATAAAATATACAACAAACAACGGTGTTAGAAGTGAAGTAAACAAATATGGTAAATATAATCAGACATTCAAGATGTACTCGATAAAACATTAAGTTTACATAACAGCTTATTTCGGAGTTCGAGTTAGAGAAACTTCGTTATTTGGATTACTTTTTGAGGTATGTTTAACATATATTTTTATATTAGCTTATAGATTTATATTAAAACATTTGTGTATTTGTGTGCGTATTTCCAATCAGAAACGTGCTCACGAAAATACACGTTCATGACTTTTGCGCTCACGAGCACGCATCAAAAAACACATCTCTCGCGCCAACAATCCAAACCACCCCAGAGTTGCGCTCTGTCTTCTGACACTGCCACAGCACACAGACGGCACACAATGCAATATGTGTATGTAGCGGACTCGTTTTGGACACGCCCCCCTGATTGGTGGTTGTGGCGCTCATCTTTGAAACTAGTCGTACAATATGCTCATTCTTGGCCACTCCTTGCGACAAGTGCGGACAAGTTTCAGTGCGTTCGGTGCTATTGGATGAAATGGTTGCTTAAGAGTCGTGCCCGCACGAACCAGCATCGTATTGCCCAGAAACGGAGGGAGAGTTGCGAGTCTGAGTCTTAACGTGTTTAACTGAGCAAATGCATGGGAAAACAATCTAACAATTGAAGCAATTTGTTGTGTCGGAAAAGTGCAATAAAATAGTGAATAATACTAAGTTGAAAATAGTAACAAATAACAACGGCAAAAGCCACCATTGAGGCAACAACAGCGACACCAACTCAAACCGGTTCTGCGCACCGATTCGCGAAGCTGAACCTATGCCTCCCAGAAAGGGACAGCTACACACAGCACTTTAGTGGCGCGAATGTGTAGAAAAGTGTAACTTGTAACTACAAATTGCAGTGCAACTGTATGTGTGTCCAGTGTGACTGTGTTGTGCATCTGTGTGTGTGTGTGTGTGCGTGTCTCTCTCTCTCTCTATCTCTGAGTACTGTTTACTGTGTACTGTGTGTTGGCCATTGCAAGATCAGCTTCGGTTTCTTGTCACTTCTGTCCAGGCCCTTTACTCAGAGCTCGAAAGCCAAACTCGAACCTAACTTTTTTTTCGCATACGCACCGTTGTACCGGGTTTTGCATTATCTTCGGTTGTTTTGCGTTTGTTGTGTGTTGTTGGTGCTCCCATTGTTATGGGCACTCGCGTTGCCATGGGTGCAAAGGTGTGTAAAACTGTAGCTGGCAGCTCGACATGATGGAAATGGAACTGTGCCGCTCGAGTAAGCAACGCTCGACACCACCATTGCCAGCGCCAGAGCCAGCGCCAGCGCCAGCGCCATCGCCTTAGCCATCGCCATCGACATCAACATCGCCATATTCGTAAGTTCGCGTCGCGGTCGATGACAACTTTGTGCCTGCTTTGCGGGCTTATCTGACCCTGTTCGTAGAAACATCTCAAAGATACATAATAATATACAGTACTTACGCAGACAGAGAGTACTCAATTCACATAGTTTAAATGCTAAATGTGGTGCTATTGTGCACATCAACAAAACAAAATAAAATAAATGTAGAAAAACCCGCATTGTGAATAGGTGCTGAAAATGTGAAAAACACAAAATGCTATTATTTTGAGCAAACGCTGCGCATGGAAATCACTCAGCGATCTCTTAGAATGGTGAGTGAAAAGTTTTCTATTTTTATTTTGTCAACTTTCGTTTATTTCTTGTCTTTCTAAAAAAAAAATCTTGTTATAATAATAATATAATTTACTAAACAACAAACTTGTTTTGTGTATCATTAGTAAATTACTTAATTCTCGAATAATAATTGTTGTAATTCAACGCATGTTGCATTGACTTCCATTTTGCAGAATGTCAGACCTTTTAAAGCAAAATATCACTTAAATAGAAAACAACTTATGCTGACCAAAATTATTATTGTGGTAAACTCATAATGCCTATTGCTTAAATGTTTTATAAGCAAAATTTAATTTGTGTTGTTATTAGTATATTATTTATTGAATGTATTTCCAATAAATTCTTCGTGCTACTATTGAGCGACTTTCAAATAATAGAATTATAATTTCACAGATAGATACGAATATTGCTAATGGATTTTCCGACGGCCATATTTTATTTAAGAAGAATGTTAAAGAAAACCATTTAATGATTTTTCTAACACCAACACCCGTAAAAATAGCATTGCTTGATTAATTTCTTTTTACTAGTTTCGAGGACAGGTTGGCTTAATAATTAACTATGTAAACACATTTTGACCATGCCGTAAAGCCAATAAAAATAATAAAGTTTTGTCGTTACTCGTGCCAAAAATATTTGTGAATGTTGTGCAACACACTAAAGTATTTAAATATAAATAATGACTGTTGTGCAGAGATGCAATTTAATTTAACCATTTAAATAACGAGACATAATATGTCTAAACTAAAATATATTTTTTTATATATTATATTGTCATTTTGGGGATTGATTATTTTAAAATATCGGTTCTCCATTCCAGATGCATGACTAAAAAGGAAAGCTCAAGCAATATATATAGAGAATAGACAAAAACAAGTGTAGTTTACAATGCAAGAGGTGTGCAGTACATTGGACACAACCCAAATGGGCGCCCAGATCAAATCGGAGTCGCCACTCAATCCGCTTCAGGTGCAAACGGGACAGACGACGGTGGTGCCCGTTGTGACCGGACCGGGGCCGCAGGGACCGCCGCCAGCGGTGATGCTTGTCAACAAAATGGCACCGAACTGTGATAAACGAGCGGCGGACACCGCCTATTGGATGGCCTCCGAGGGGGGTTTCATCAACTCTCAGCCCTCGATGGCCGAGTTTCTCAATCACTTGAGTCCGGAGAGCCCGAAAATTGGCACACCTGTTGTCGGTGGCGGTGTCGGTGGCGGATATCCGGTCGGTGTTGGTGGTGTACCGCAGACACCGGATGGGATGGACTCGGTGCCCGAGTATCCCTGGATGAAAGAGAAGAAGACATCGCGCAAGAGCAGCAACAACAACAATCAGAGTAAGTACACACTTAGTAAGTGCCACTTAAAACTCTACGCTTGCCACGGCAACATGAAAAGCCTCAATCAACAAAGCAACTGAAAATGCAAAATTCTCATTAATCAAGAAATCAGTGCAATTTAGCTAGAAATCGACCGGCGCCGCAGCCCAGATTCCAATAACAGTTCCAGTTGCTCAAACCAGAACCCAATAAGGCAATCCCAGCTGGCCAATCTCATCTGTGGAGAGCCAATCTGAGGCCCACTCGATCACACGAGCACACGCAACGCGTCTTCGTCTTCGTCTTCGTCTTCATCTCCCTCTCTCTCTCTCTCTCTCGCTCTCTGTCTGTCTCTCTATTTCTATCTCTCTTCATGTTTCTGGGTTCGGCTCCTTTGATGGACAGCGCGCCCTGCACCAAGCCCGAAACCGGTGCGGCAAAGATCTGTTATTTGCGATCATTTGTAACAATTTTCGAGAGTGGCCCGACGTCGATTCGTCAGTGCGACTGATGAATTCAGCTCAAGACGTTCTTTCGTTTTGTATTTTTTGTTGCTATATTTTTTAACCCAATGTGTCGATTTCATTTGTGCAGCAGAAACTGAAGCCAGCCTGCGACTGTGGCAGCTACATTTTAAAATAAATGAGGTATCAAGGTTGTCTGACCATACGTTTTCCTGTCCAGGTACGACTGTGGGTACGAGCTGAGGACCATGAGAGGCCATGGTCCAGGGGTCCAGGTTCCACGCATATGTTGCACACACTCGCTGTCTTCCCTCTGAGCGATTGCAACCTGTTATTTAGGTTCATTTGTCAAAATATTTTTCCTGTCCCCTTCTTCGATAAAGGGGCTGTGACGGCAATGGCAGGAAAACAGCCGAAAGCTGGGAATGGGACCAGCGGACTGCCAGCAACAAACAACAGTTGCAACAGTTGCAAGTTGCAGTTACATTGCTACACATATGTACACACACTCTCTGCAAATTTTGTGCCGAATCTCGCATTTGTGCAACATTTCGAGCATTATCAACATTTGCCATTCCATTTGCCATTCACTTGGCCAGCATTTCAATTAATCACAAGTGGACACAAGTGGAGCGTTTATAATGGTGCGTTCACTTATGTTACTCGCCCATGGCACTTGACTCGCAGTTGCATTAAGAAGGATGGTCGACTTGGGCGCAACCAGTTATGGGCCAAAGCCAAAGTTAGCAATAACTTACCTCTAAGTTCAGCTTGTAAAATTAAATCTTACGAGCACTTTTAGATACTCCCATTTTTAGAAAAACATAGCTAGTGGCAATTTAAACCTTTAAAAAAAATGGTTAATTAAAATTTGAATCAGACTTCAAGTTCTTTGTTGTTTAAAATAAATACATTTTGATGTCTTAAATTATAAATCAAGACTAAAAACTGAACTCAACTATTCGATTTCACACCACCAAAATCATATTTCCAGCAATTAGAAACCATTACTAAGCATAAAAATTAATTAAAAACTTTGTGATTTATAATAGAGATAACAAAAAAAAATAAAACTCAGCGGAAAATTCGCTATGTAATCAAATAAAATTCGAATACTCTTCATTAAACTCAAGGTTAGATTCCCCGCTGAATTTACTCTATGTACGTGTGTGTGTGTGTGTGTGTGTGTGTGTGTGTGTGGGTTGCATTGACCAACACTGACTTTAACTAATACAACGCCAATTAGTATCAAGCACACCATCATGTTGACAAATGGGAACCACTAATCCGACAAATGTTTCAAGTGTAACGTGAAATCCAAACACGTGGCCAGGGAAAGCGGGAAATCTCTAATTCCCCAGCGTACGGTTGCATTTTCTTGGGGGCAGTTGTGGGCGACACTGAAGCTGCCACACACTCACAGTCCGAGTCAAAGTTACAGTTACGTGCTGTGGCAACTGGGGCCGCTTGAGTCTCTTTCTCCGTCTCCAAGTCCGAGTGAAAGTGAGCCAATAAATAGCGTGAAGCATTCCAAGCGCCGACACTCGTACGGAATCTGTTGAAGGGTCAATACATCTTTTCTCACACACACACACACACTCGCATACACTCAGTCGATTGGGACTACATGCTGAGCGATATGGATTACAAATGATGTTGTAAATGTCCGTCCGTTTGCCAAGCGGGCGGGAAGGCCAGAAAACTGGGAACTGGGTTCTGGGAGCGCTGTTACCGTTCTCCATTGGCTCGACAAATGTGTGTGTTTGTGTGTGTGTGGTTTGTGTGTGTATGTTGTGTTGGTTTAACAGTTTAATTTGTTAATGTTGTCAAATTAATTCCTTGTCAGGTTCTGTCTGTGTTCAAGTGCTGTCAGTATCCCACCGAGTGGAGGATCGTTAAAATTAAAACGTTTCCGGGATCAGTTAATATTTAAAGCGGCTAATTCCAATTAAGATTTGCTCAGTGTAAAAATCTTCTAAAGTACTGACAATTATAGAACTTATATTTTTAATAACATTCTTATTACCTTATCTATTTGAATGCCTTTTTATGTAATACTAAAATATAATAATATTATTACATCCAAATCTAAGACATATAACCCTTTTTATTTGGAAATATGAGAGCATACACATTTAATATTGAGAACAAATCATTTTGTTATTCCGAGAATGTGTCTAATTTTGTAATTTTTCATATTCTCAACCTCTTCTATTTCTAAAAGACTTAAATCAAGGCTTGTTGCGCTTACCGCAAATTATTTGAATCACATCCTGAATTTTAATGTGGTCGCGAGCAGAGTCTGTGTCCGTGGGTGTTTTGTACCCCGTTGGGCTGTTGCTTGGTGTGTGTGTGGTGTGTGTGTGTGTGGTGTGTGTGGTGTGTGTGTTGCTCAGTGTGCGCTCTCAAATACAAATTGTTTTGTCAATTGGCTGGTGCCTCTGGCGCTCACTCACTCACATAATTGAAACAATAAAATTTCAACTTGAATAAAGTGTTAATTAGGCGTAAAGCTTCCAGCTTGTAATGTACTGAAGGCAAAGTTATAACACGACGCGTTTATGGCTTCGCAAGATGCTCAGTTCGCAAAGTATCTGCGAGATACGCGAGTGTATCTCAACTCATGCCAGTTGGTTGTTGTTGCTGCTGTGCTTGTGTGTAGATAGGTGTAGGTGTGTTCATAATTGTGGCTGGCTTTTAATCCCAGTGCGGCCATCAGTTAGCGAAACCAATAAAAAAGCAACTTGACTTGGTATTCGCCCCCTTTTTGGACGCGGCTATTGAATCGCAATCCAGATAGATTCGTGCGCCATCTGTATAATCCATCCACCCACCAACCGACCATATGTTGCGCCACTTCATCCTGCGCCTCTGTCAGTTCCAGTTCCAGTTCCAGCCCCTGTCTGAGGTACGGGGGCGTCGCTGCATAAAAAATGCGTAGCGCACGACTCGAAACTTACCCCCTGCGGATTATGATTATAACAAACAATTTATCATAATATTTATGTTGATGCGTTGTACTTTTTCTCAATGAAGGTCTCTCCTGCCTCTCCAGACTCTTTCAGCTTTCGGCTTTCGGCTTTGTCCGTGCTGTTTATTAATTTTATTTATGATGAGTCATTTGCACATTTATAATTTTAACCTTTACAGTTGAATTCATTCAGAACAAACCGCGACCAAGTGCGAAAAATCAATGGAGTCTTGAAGTCAATGCATAATAATACCATTCAGGAGATGTAAAAACACATGTATCCCTGTGGATGCAGCTTAAGTTGAAACAGATTTTTGGCTTATCTTGTATATGTTACTAAAGCGGATTGTAAGCAGGGAGTAATCGTTTTTTATGACTTCATTAAATGAAGAAGAAGATTAGTCTTTATTTTATTAAGCTCAGCAAGTTCATATAGTATTTGGGTAAACCGTCAAATAGTATTGAAAATAGATAAGACTTCGATTTGAGAGTGGTACAAATATTGCAACAAATAAATGAAAAAATAAAAAAGTTACAACATCATGCCGCCCATAACACATTTATTTTAAATCCTCTTGCTTGACAGTTGAAACAAAAAAAAATCATTAAATTAATTGAGGCTCGTCAAATAATTTTTAAGTAATAATTATGCGTCGATCCACAAAAGGTGATTTCGTTTTTTTTTGTTTTTAAGCACAATCTGAGAGACAGTCGCCTTTTAAAGGAGCTCCAGATTGGGTTAGGCCTGAGCCACTGAAAAGGTCGCGCAATTAATTGTTACGACTTTATTATGGAGATAAGTGCGCCAAGATACTGCGCCGTCTTGAGGAGTCCAAGGCAAACAAAGGGTTCTAATTGGCAGGCCGCAGGATATGGCCAGGCAGACGTTTACCCCCAAAGGGTTCCCCTTTTTTGTGCTATTCTCTCGCATCGTGACTGGGAAAAAATCTGTTACGATTAGTTAAGCTCGGAAAAGTACGCTGGCGGCTGCCCAAAAAGAAAACAAGTCGTTTTATTTTTTTTTTTGCGGTCAGTCCTTATCTGCAAACTGATTTGTCATTATTTTAAAGCACATTCTTACGAAAATTATGAAAAATTAATGTTCATGATACTTGCGTGGTACGATTTTCATTCTACTCCGAAAAACACACTTATCCAAACTAACACTTTTATTTTAATCAAAAGAAAAATACTCTCCTTGGCACAATGTAGTTTAAAGAACATAGTAAATTTAAAATTTATGTTTTTTTTCTCAAATATAATTATAATTTAAGAGCATAAAAGCACTAAAAATTTTAAGTTTGGTCAGGATATCTCGGAAATCAAAAAATCGTTCATATAATAACTATTTTTCAACTCAAATGTAGGCTTTATGATTTAAGGTTATCAACTCTAAAAGGTTAACAACTGCCATATAGAGGAAAGAGAGCAACAAATCGATCTAAAATAAAAATAAAAGGTTTCAACAAACTCCTATTAAAGTAACTGTAATGCGTACCCTTAGATCTATCAGATTTTCATATCGAAAGCATTAAATTCATAGATGAAACCCCAAATATATTTGTAATTTCGTTTTATTACGATGATATTCATCCCACTGTGAGACTTGGCAATTTAGCGCCAGCAAATTAGGAAAGCTTTGCGAAAAACAACTTTAGCTATAGAATTAAACAACGTCCAGTATCGATTTAATTTTATTTCCGCTCATTTAGCTAAAAGCTAAAAAAAAAAGTGAAATGAAAATATTTGTCCACGATTAAATTTAAAAAAAAAGAAGCATTTTATGAAATGACTGCACTGACACTGGCCTCGCCTTAGAAAATTTGAATACATTACGAGCTTGGGCGGCAAGTGCGGCTGCCCTTGTTTGGAGCACTGCGGTGGTTGGTTTGCCAGCCCCCTGAGCAAATAAACTACGGCAGTGAAAGCCGTGGAAGTTGAACTCGAACCACATCCCCATTGACATTCCCATCACCCCATTCCTCCTCCCATCGCCCCTTCCATACCCTTGGCCAGGACGACGGCATGACAATGACACAAAAGCCGCGCACTTAATCGCCGCGGATCACATTCCGTAAAAAGCGCGACGATAAAATTTTTTACGCTCTATTCTTGTGTACTTAGTCAAATGGAAGGGAACACCCCGCGTTGTGGTTGCGGTGGTTTTACCGCCTTACCCACTTAGTTTGACCCGCTGTCCACATTTGAATGTCTCTGTCCATTTTCAGCTGCTGCTCGCAGTGTTCTCCTTGTTGCTGTTGTTGTTGCTGTTGCTGCTGTTGTTGTTGTTGTTGGTAGCCAAGCGCGTTTTTACCATCATCGTAAAGAAGAAAATCAAATCTGAAATTATTTTCAATTTGTACGCGATTTATTTTTATGTCTTTTTCGCTGGCCCGATCTCCTCGACTCCTCGACTCCTCAACTGCTGCAGTGTACACCCAGTTTAAACGATTTGTAAACATTTGAATTGGATTTTCGGCCATAAAAATTGCTTGTTAGCGGCATTGTTGAGCGTTTATCAAAAGACCCCGAGCCCAGCTACGACTAAAGTTGTCGACATTAGTGGCAGCCACAGTGAATACAGCAAGTGCTCAAGTATAAATACACCTATTTATTACCGCATATCAGACCGTTAATCGCATTACCCACACTCGGTTGCATGCGAATACACTTGCAATTGTCGGGGAAATACTTGCGACTCTTCAGTTTCCAGCACTCAACGATAAGTTCTCGTCTGTTGCAGCTGCATTAACAACTGTTTAAAGTTAATAAATTATCGGATTTTTACATGAAATGCCGCTTAGTTATCAACGAGACAGTGCAGCAATTAGTGCACTTAATGGATGGATACAGGGAGCGGGTTTTGCGGAACACCTTAAGGCAAATGAAAGCTAAAAATGCCATAAATTACTTATATCATACGTAAACAAGTGACTCTGATTTCGACTAGTCATCATTCACGAAGTAACGCTCTGCTGATTCTAATATTTGCAGACCTCGAAAACTAATTAGTTGAATACAATCGACCGAACGAAATCACAAGTGTGAATTTTGACTACATTCAGAGCAAATATTTTTATATGTAGTATGAAAAATCATAGAATGCTGTAGTCGAGAGAGCTCGACCCTAAACCCTGCTGTTGAATAATGTAATAATATATTTTGCTATAAAGTAATATGTGTTTTTGACGGAACCACTTTTGGTCAATAAAATATCTATATCGAAAATATTAATAGATCGATATTCACCAAACTGGGACTGTTATCGATCAAATGAAATAAACTTTGGCTAAATTTGGTATTTGAAGTGCCTGGATCAACTAATTCATACAGCATAGCAACTATACCTAAAGTCATATTAGTTGCAGTCTCGATTTGGGTACGAATATATCATATCTATGCAACATTGATTTGTTCTCAGACCATTTAAGAAATACAATAATTTATTTATCGAATTTATATCCATTTGCTGTGGCAAATGTGATAAATGTAGCCCGAAAGCCAAAGTTCGGTAAGTAGACAAGAAGCGAAGTCAACATTGAAATATTTAACGGCGTTTAAAAGAGCGTGAAAAGTCAACAAGGATAACAAGGACAACAGCCACACAAGTATGGGCTGGAACACATGCTCGACGCTGCTCCTTTGGCCTGGTACAAAGGTCGTGGTCGTGTGCCCCATGTGTGTTTGTGTGTTTGTGTGTGTATGTGTGTGTTTGTCTGCGATTTAAATAAAACTAATGTTTGTGTGAAATGGATTTTCTGTTTGTCATCGACTGACGCAGGGGTGTGTGTGTGTGTGTGTGTATATGTGCTTATGTGTGTGTTTGTGTCTTTCCCACTGCTTTATATTCAAATAGCGAACCACAAAAAGGCTTCAAGGATTCCGTATTCAAACAGATTTAAATTCGCATACGCTTAGCACGTGAATCCTGAATCCGAAACTCGAATCCTTAAGTCTGCCATCGAGTTCGCCTTCCGTCGAGTCATGCATCATATCAGCTGCGGATGGGATGCGATTGGATGGGATGGGATGTTATGGTATGGTATGGTATGGTCTGGGCTGGCATGGGCTGGGGGCATGCAGCGCGTGGATGACACGTGATTCCGTTGGTCGTAAATTTTGCAGGCACGAATTTTTAGTGCTTTGTAGTTTTAATTTGTGGCTTGTTGAAAATGTTAACAGAATTATTTATGCGATGAATGGCGCGATAAAAATTATGATGCTAAAAAACTTTTTGCAACGGATATTGCCACAAGGTAGTCAAGCACAACACGTACTACCTGAGCGAGAGATAAGAATAAGTGTTTAAACCGAGCTGGAATTTGCAACCACCCAACAATTTTAATAGACCTTTCAGGGCCACAGCACAAAATGTCACTCAGCCATTTCATTCATCTCCGCGTCGATTGGGGTGTCATGTGAGTGTCTGTGTGTGTGTGTGTGTGTGTGTGTGTGTGTGTGTGTGTGTGTGTGTGTATGCAAGTGCGAGAGTGTGAGAGCGTCATGGCATCTCCTCATTTCGGACTTGACCTTTTTTCTGGCTAAGCTTCCGTTTAGCAGGAGAATCTTTAATTTTGTACATTTATCTGGGTGATGAGGTGCGTAGCAAGCGGGGCACACATGCTGCAGACACAGATACAAGATACAGATACACACGTTACGACTTTTGAGTTATTTTTTTTGGGGGGCCGTTGACAGCCAAACGTCACTTGGCACACAGAGTGCCAGACCGTCAGACAGTCGGCCATGCCACATGCCGCATGCCGCATGCCACATACATAAGCGACTTTTGCATGTGGTGGTTTTTACTGGCGAAATCTGCACTGCAATGGTTTATTGTAGACGTGGCGATGATTATATGGTTGTTGGTCCATCATGTCGGGGTTTCACTCTGGGCTGCGTGTTAAAAATGAATTTGTCATCTCTTAACTGTAAACTGTGTGACATGTTTGCGAAAATCGCCTGCCAGCCAGTCATCATTCAGAAATCACAGACCCGGCCAATACATCAACTGCTTTAGTCGATTCGATGGCTATGGTCCAAGTCTGATTGATTTGCATCGGGAATTCTTATTTAAAGTGACATTTTTATTTCGTGCTGCAAGCCAAAAGTTGGTGTCGAGGTTCTTGTTATTATTATTAAGTTACTTTGTTGCCTTGCGTCAATCTGCAATAATTATGTCAGCAGCATTTATCCATGTGATTGATAATCGATGATGCAGTTCATCGGCTGATGGAAATATTCTGCAGTAAATACGCTAGTGATAAACAATTTTGGGATAATTAAGAGATAGCAGTGTTATAAAAATGTTGAGCAAATTTTACATTATTTTTGCAATAACATTTTATTCTTTTCCGAGCATTTGAATATTTTTAAAAATTAAATATACTTAATATAATTTGTTAACTCTCAATTATAGACAAATATACATTTGTAAAGCTATAGAATATATATATATATACTTCGCTAAGATCGGATGAGAACCTGATCTTGTTAAGCAAATTAAAATCAGCAAGGCCATTAAAAATATAGCTTATATGTAGTCATGTACAATACAACATACGTCAATTCGCAGAACAAATGCAGCCATAAATTGTATTGCATTTTATAGATACATTCATGGATAATGTTGACACATTAAATCGAGCGATTCATTGTGCATAAATAAATAACTGGCAGTTGAAAACACAGGCAAGTTCAAATGCAATTACACACTTGAGTATTGAGTATCGAGTGTAATTACAACTAAGCGATGGCGGCAGTTATTAATTGATATTCGTACATGGACAACAGTTAAATGAAATAAGTGGGCCATTTATGAACCATCAAACAATTCCAACTGTACTCACATATATGTACATATATATATACATTAGGATCCCCGACCCGCTGGCGTTTACCTCCATGGTTACATGCTCCCATGCGCTGTGTAATTGGGGTGTGTTTAGATCCAATTTTAAATGGCAATTGGCATAAAAATGTCTGTTTGGGTGGGTGTTTATAATAAACGCATCGTTCAGATCATTGGACAAATTGCTGTTCGAATAATTGCTATGATTTTGCTAAAGTGCGTAAAATTGTTATGCAATTAAAATATAATATTTTTCCACATGAGTGCCGACATAGATTATATAATAATTTTAGCATCGATTTCAGCTGAAGCACGTTCCAATCGCAAATGTAAAATGAAAAACAAGAACAAAAGTAAATCATTTATTTGTTCTGGCCTGAATTTGGAAAAGCCCTGACTTGTTTGAGGGGAAAACAGCTGTCGCTGATTTTGTTCGATGTGCGTGAAATATTAATTTCACAGCGCGATTTTTAAATACAGATATTACATAATCGAATTTCGAACAGAGCCGTATATTAAAGCTACGGCTCAGCTATTTTTCTTATATCCCGTTTAATTTCTGGCTGCAAGGACACAACGTCTGTCAACAGCAAAAGCCACAGCAGCAGCAGCAGCAACAGCAGCAGCAGCAGCAACAGCAGCAACAGCAGCAGCAGCAGCAGCGGCAGCAGCAGCAGCAACTGCTGCGGTTAATGGCTTTTGAATGCAAATTCAATTTTATCGCACTGTTTACTTTTTAATAAGATTTCTTAACGTCTGTCGCTGCGACTGCCGCTGTCTCTGCTGCCAAAAGGTGCACTGTGCTGGAAATGAAAATTAGTTGCAAGCGCAGCCCACAAAACCAAAAAAAAAAAAAAAACAAAAAGCGAAAAGAGCCAAAGAAAATATTCAAATACTCCAAAGAGGCAAAAGAGACACGCGACTGGCAACTGCGAAAAATGCTGTTTGTGGCGCCACAAACAACTACCCCCGCCCAGTCAATGCCATTGCAAAGCCTGTAGGCAGCGGTGGTGGTTCACACACACACATACACACACACACACACACACACACATACACACATCCACGCATACACACATACACTTAGATATGGCAAGCACTTTTACCTTTCGTGCTTAGAGGGCACAACCTACAATCCAACAAATTGCGTTTTGTGCAAGAGTGGGTGGGAAAACTGTCAGAATGAATGAGTAGACTGGTCACACCTACTGTCAGTCAGCTACGAGGCAGCAACAACAACAATAACAACAACAACAACAACAACGGTATCATTTTGAGCTGACCAAGGCGTTGCCATTGCTCTGCGTTTGGGTCTGCGTCTGGCCAGAGCCAGAGCTCTGGGCTCAAACTGGCTCTACTTGGGCTCCACACAGCACTAAAAACAACTTTAAAGTTCACTTACATTTTTACAAATTTGTCGAATTATTTTTTCAATTAGGCAAATGGTCTTTAAGCATGGCTGAAAATCAGCTCTTATAATTAAAAAAAAAAATCATTTAAAAAATTCATTTATGAAGTACATTGCTTAATTTTATCCTTGAAAAAATAGGAGATGATGGACTTACTTCACAGGTTTCCTCTTAAGGAGCTGAGTCTATATCCTATATTTTGCCATAGTTAAATAATAGTATACACAGATTATTATTATATTTGCTTAATTTTTTTACACGATTCGAGTTAGTTAATGACTCACAACATCTTTAAACTCAATCATATATGCAATGTTTAAGCTGCAAGCAAAATAAGCAATTCTTAAATTGCCACATCAAAATGCCAGTCATATGCCTGAGTTTGGGGTAGATGCAGCAAATATAAATACTCATATTTTCTTATTATCTATCGGAATAGTTGCTAATCACGCAGCTGTTGGCCGTTGCTGGCAAAATCTCAATATATTTGACTGTTTTGCTTGTGAAACTGCAGAGCTTGGCTCCACTCCCCAAACCAGAATTAACTGACAGCCCAGACGCTATTATTTCCAACCGCATGATTTTATTTTCTCGAGCTTCTCCCCCTCGATTCTCCAACCCCTGGTGGTTACTCCTTTGATATTTCGGTTGGGCGCTTTCCTCACTTTTGTGCAAATAATGTACTGGCAGACAGGAGACAACCGCCTGCGAGTAGCTTAAAGGTTTTTCTCTACTATCGAACGACCGAAAACTTCTCAATTACATGGCTCGCAAGTTGTTTAGTATTTTGCTGATGTCGCTGGCTCCGGAAGCGGCACGGCCACTTTCTTGACCGCTCGTTACGTGCTGCATGTGTTGCCCCATCCTCCACTCTTGCTCTGGCAGTGGCTCCAACGCTGGCTTCGGCTCTTGGACTCGGTCTGGGCCCAGCTCTGGGTCTGGTCACATGCAGCAACAACTTGGAACTTCCGCTACCAAGTTTGGGGTTAATTATTTTCCACTTTTGTGCTCAATTTCATAATACTTACTTACGACTAAGTAAATAAACTCAAACTTTGCTATTTAAACTCCGCACCAACTTTCTTATAAGCTAAGGCACTTCCAACGTTGTCTTATTTACTTATTTGTTCGTATGAGAAGCTAATTTCTGTACAAATTGTGACCAAATCAATCATCAAACATTTGGGCCAACTGATCTAAGATTTCGGAACAATAGGACAAGGCTTGAATAGGTTTAAAATATGTATTTAATTTGCTCTATTCGAAGCAACCAACCTGTTTTTAAAACCAACCTACCTCAGTATAAAACAATTCTTAAACTTAATAGTTAATATAGAAACATATAATTTTCTTAATAAATCAAAAGAGTGAACATTTAATTTTCAACTCTAACTCAGACTCAATTCATAAACGATTTCGTTTTCTTTCTACTTTCAGGTGATAACTCCATAAGTAAGTACAAAATACTTATACTCATTGCGCAACATCAAAACCACAACATATCAAATTGGCTATATAGATAAGTACAGCAAATTGCATTCGTTTGAGTTCAAAACGTTCCCAAGATCAAAATTTCAATAAATAATATACAAAGCTACAAAATAAAATAAAATAGGAATAATACAAAAATTGGTTTTGCAAATGATGGCAGAAACCGCAACATTATTAAATGTTATTATGATAAAAGTATTTTTTCTACACTCCCCATCTTAAAAGTGTGCGAAGGTTACGAAAAGAGCCGCAAATGAAACAGCTACCTTTTTAAGCCATTTCGTTTTGGCAATTTGAATGTTCCATTTTGTAGTTTCTCAGCCTCGTTTTGGTATTATTTTTCATGGGCCAAAACAAGCTGCAAAGTCAAGATAAAAGCATTAAGAAGTGAAATTATTATAAGCATAATTACATTTCATTTTTTTATTTTTGTTTGTTTTTTTTTTGGGAACTGCAATTTTCCTTTCATTTTTTTCTCGGTATCCCATAAGACATTTTGCTCTACTCGTATTCCCAAATTCATATTCGGAAAGTTAGTTGGCTGCCATTTTGAATTGGAAGCAGCTCCGTCTAGACTCTAGACAGCGGGCCTCAGCCTGGGGCTCAGTTAAAAACTGAACCAATCAACCGTCAACCATTTTCGAGCAATTCGACGGAAACGACGCTTTTTCATGTTCGTGACCAAAGCTTAAGCGCGCTCCGGGCATGGATAAAAAATATTTTACCTGAATTAAATTGAGTAACAAAAACACGAAACACCAACACACAAACACACACACACACAACTTCACACACACCCATATATTCTTGCACACACACATACATGTGCATATATTCTTGCATGTAGGTCTGTGCCACATACATATGTGTGTGCATACATTTGTTTGTATGTGTGTGTTTTTTATCTTTGTGTTGTAATTTTCTACTCTTGCTGAAGGCTAAGTTACTTTGTTATGAAGTATGCAAGTGTTTTTTATTTATAGCTGATTTAACTAGTTTAATGGTTTGAAAAAGAAGTCGAATCTGCTCGTAGAGTAAGCGATTGCAGGCCAAAGCTCGAAATATGAAAATTATACAATAATGGCAACATTTTTAATTTATTATTATATTTTATATTACTTTCAAAGATCTGCATGACATTTAGAGACTTAGAGCCAAAAATCAAAAGTTTCCCGTGTAAAGTTAGCGTCGACTTGAAAAGTTAAAATGTTCCATAAACTGTACAGTCTGTGATAAGTTTTCATGTTGACATTTTGTGTTTCTGTATTAGAGTATAGAATCTTCGGCTCTATAAAAGTGATTTATTCTTTCTTGATTCTCCAGTTGTTCTGTTCCTGTTTTGCTGTTTGTTAAGCATAAAAAAATGTACAAAGAGAGAACAAAACTCAACGCGAAACACGCTACCAGTTTCGGTTTTTTTTTTTTTTTATTTATTTAGTTTTTTATATCCAATGCCTTCCAACTTGGCCCCACTTGTAATATCATTTGTCGATGTCGATGTCGCTGTCGCTGTTGCTGTCGACTGCTCCGTGCATGTGTTGGTCACTTTTCTGCCCGGACGCGTCGGTATTGGTAAAGCGGTTGCGGAGGAAATAATTACATGATTGAGGCACTCACACACAAACGCACACAAACATACACAATGACAAAAACAACAACAACAACAACAACAACAACAATGTTCACCTTCTTTGTGGTGGTGGTGGTGCTGCCAACGTCGTTGTCTGATCGAACAGAACACGTTAGCCTGGTCAGCTCCAGCTCCAGCTCCAGCTCCCGCTTGTGACCATTGCATGACAGGGTCGTGTGTGTGTGTGTGTAAGCACACGGCGAGGTGTGTGCGTGTGTGCATGTGTGTGTGTTTGCAAACGCCGGCTCTGGAATGAATGGCGGCTTTGTATACTGAATTCTTGTTTAGTTGTACTCATTTTTGTTGCTGCCACTCTTTTTTCTCTTTATATTTATGTGTATAGTTTTATTTTTTGTAATTCATGCCAAAGCCGCAACAATGACGATGATGATGTGAACGACAACCCAGAGCAATTATTGTATCGCTTGCCAACAGCAGCAGCAGTAGCAGTAACAGCATATGCTCTTTTATCCTGTCCCACCCCTTACCCGTCATATACCACAGGGTATACGAGTCTTGTCCCATTGGCTAGTTGCTCATTCGCCGAGGCGAAGGAACAGTAAATACACTTGTGTCAGGAGCATATCATATGTAAATATGTACATAAGTTGACAACATGACTAATTTTTCGAATATTAGTTATGTAAAAACATACAGTATGGAATATTAAATATATATCAGTGAATTGGGACATGGTCTAAATTTGTAAGCCAGATAATGAAAATGAACTCTTGCTTATTTCAAATATAGCTGTGCTCTTAAATCGTATCAAAATCATAGATTGGAGTAAGGGACTTACATCTCATATAAGTTGTTCACCTTCTTAAATCAATAAATTAAACTAAACCACATCAGCTTTAATTTTGTAATTATGTTCCTAAATTAAGAACTTGAACGTTTTGTGCTGTGGGTGTAAACAGTCTGTTAATTAATTTGGAAAAATACTTCAACAAGATCTTCTTTTTCCATTCCCTTCCACTTAAAGGTGTGCAACCCCCTCAGCAGATTCCTTCCCAGCTACGATTTGTCTTCTTCTTCAATTAGTCTTCCCTTAACTGTAGCTAATAGTTGGTAAATAGTTAGCCAATATTTACATGTGGGTTGTGTGGCCTGTGGGTTGTGTGGTGTATGCTGTGGTCGCATTATGAAAATATTTGTCTTCAAATTGCTCATCTGTGGACTTCATTGTCTATCAACGTGTCGTCGTCGCCCTCGTGCGGAGGCCTGCTGAGCGTCCGTCCGACACTCTGACTCTTATGGCCGGGCCGCGACGCCTGGACGCGGCAGACGTTAGTCTGAGGGCTTTCAAAACGTTTTCATTACAATAGTGCGTAGCCGACGCCGCAGTGGCCGCCGCTGCTGCTGCCGCTTCTTCTGCTGCTGCTGCTGTTGCTGCTTCTGCTGCCGATCGGGCAAATGTGTCAGAGCTGAAACGTCATGATTAATGATGGTAGCAAGCATGTGGGCCCGAAGGCCAAGCTAGAGCCACAGCCCTAGCCAGAGACCACGGACTGGGCTGCATTTAGAGGCCGAAACGGAGACGGAGACGGAGACAGCCTGGGGGTAGGGCAGAGCTAACTGGCAACTGGGCCATGGGAAGGGACACAAAACACTGGGAATGGGAATGGAAATGAAAATGGGACCACGAGTGGCACTGGGATCATTTTATCAAGGTAAATTAGACAAAAACATAAAAACACGACAACCAAAATGTAAACAACGCACCAAACACTGAAACACTTGGCGGCGGAGCATTAGGTGAAATTAGAAATTAATCCTTGTGGCTGCACTCGTTGGAATTGTTGTGCCTTGACCATGGCAACAACTGCTCAAAAGGGGGCGGTAACATGGCGTCGATGGTTACTATAATAGAGGCATGTACTTGCACATGCTCAGAGGGTGAACTAATGACAGTTGCAGACATATGCTTGTGAGAATATTATAAAATAATCTACTAAAAACATTCAACAAAAATGTATACAACAATCTTGAGCGCAGCTTACCGAGTCGAATTGATAACATTTATCGAAGGTTATCACATAACCAAGTGCAGTTCCTCTCAATTATGCACAGCAGCCAATTCAACATAGCAAATCAGTCATCAATCGATACACAAATCTCGAGGCAACACCAGCTGCAGCAGTTGTCTTTCAATACCATCAATTCCATCGATATTAAAAATATCCAAAATTTAAAAAAAAGAAAGAAGGAAAATGGAAAGTGTTTGCGCCTGCTTTGGGCTGCTGTGCTGCAGAAAGATGCGCATTGGCTTAATTGAAACCGTTTAAATTGCAATTAATCACCAGGCAGAGCCGACGGCGAATGTTGCACAGTTGTTGCCGAGTGGCGCAACGAATTCCCACAACGGGAGTGGTAACTTCAACTCCAACGTTTAATCTCCTGCATTTAGCTGCACAGTGGCACACACAGATAGAGAGAGAGAGAGAGAGAGAGTGAGAGTGAGAGAGGGAAAGAGAGAGGGGCTCTGAATTACTGTCACCACAGTGACTAATTTTATCCTTTGGCTGCTTTTGTTTTAATTCTGAACAAATGAAGAAGCAGCCAACAGCCAGCAGCCAGCAGCAGTTCAGGAGTCCCAGTCAGCAGCCAGGGAGGCCACACGGTAACTGTTTAATCGCTTTCGACATGCGGATTTAAGTTTTTTGGGCCACGGCATGGAAAACAGGCAGCCGGGCGCAGAATGGGAGCTGTGATAAGTGAAAATATGGATGGCAACCCAACCCCGGCAGCCGTTGAGCATGCGATGGGCGGATGAGGATATTGCCACTGTGTGGTGTGGTGTGCGTGGCATCAACCCACAACCAACCATTCGACCACCCGATAACACCACACAAAATATATATAAGTATAAAAAAAAGAAGAAAAAACCAAAACCAAACTCAAACCCGCAGCCGCAGCCGCTCCACCGGACGCACGCACAGGATGCGAGCTGCGGATGCTGGGCTGCGTGCTGAGGAGTGAGTGGTGAGGATGAGGATTGGAACTGTGACTGGGACTGCGACTGCGATTGAGCCTATGCCCATGGCCTTCTTTTCTTGCGTTTACGTCGTGCACTTCGTTTTTTAAGTGGAAATAAATTAAAAATGGGTGGCATCTCTGGAGCCAACAACTGGAGGCGGAGCTGGATTGTGGTGTGGAGTGGTGTGGTGTGGTGTGGAGCGGGGGGACGAGGCGTCTACAACACGCGTCGCAGGCAGCGGCGGCGGCAGCAGCTTTACTGGCGTTAGCTGAAGGTAAAAGTTTTGGATAAAACAGTTGAACTTCAAAATGTGAATTTGATTAGGATATTTGCATAAAATTCTGTTTCTGCGCCCGGCACTCAGCACGGGCCCAACCAGCAAAGACGCCAGCAGCCCGTATAATACCGCCATAAAATACGGTAAATACAAAACTGGAGGGGTGTCAGAACGTATGTTCTGTTCGCCGGAAGCGGAAGTATATAAGTCAACCTAGATTTGGTAGTGGCGTTTACGCTGATACAAGGACAAATGGGCTTTCAGCCAGTGCCCGTATTTCATGTGAAAAGTTGTTGAAAGCGAAAACTTTAAGAATTTCAAGTGAATGAAACTTAAAAACTAGGCTAGACAAAGCTTACTTAAAGCGGAAACAGAAGTCCGGCTATTGAGGAGATATATTTATATTATTTTTTTATTAATAAGTATCAACCTATTTAGCTATTGAATGTTTACTTTGTGATCTTAACCTATGAAAAATGTAATAAGCTTACAAATGAAAAAATAATCGAAAACAAAAATTTAAAAAGACACAAACAATCATTTTAAAAATAAAATTCAATTTGAGAACCTTGAAAGGCTAAAGTGAAATAAATTTGATCTGCTCATGGTATATGAGAATTTAAAATCAACATTTGAAGTCTCCAACTCATATAGCCTCTGAGATCGACTTGTTCAGACTGATACGGGTAATTCGACACTTCTGTCAATGATGATCAAGAAAATGTACTCCTTATGGGTTAATGGATGTTACATACTTTTGCAAGAATTCTTTGTATTATCTTTTCGCATTTATAGTTGCTTGGCAGAAATGTGAAGTAATTCTAAGTAAATTCAAAATCACATGTAGTTATAATTTCGTCACGAATACAATTTAGCTATTTTCCAGGCAAGCTGGCATAAGATAAGTGAAATATGCGCTGTACATGAATATTCTACACGAATGGAGCATAAACTATGGGACTTTGAGCATATGTCGCCACCTGTGTGTAATGTATGATACACATGCACTTGACATGGCCATGAGCCCAAGGAGCACACTCCACAAAACCCACCAAACAAGGAGTGGGAGGAGTTACAAGTTACAAGCTAGTGAGGGGTTACAAGTGGGAAAGGGGAAGAGGAAGGGGAGGGGGTGAGGGATGAGGGGTGAGGGGTTCACAAAGTGACAGCCGAAGGGAGCTGTGTGGCTCTGGCACAGAATGCAAAACTTTTACCAAGGAGCGTCGCTGAGAAGTTGCTTAGGCAAATCGTATGTGAAACATTTGGCAGCCAGCGATTTATTTAATGTCTTTGGCTCGGGACGCCAAAGCAGATGCATGTTCGAGTGATTTAGATGATTTATGTCGGGGTTTTCGGGGACACATATTTACACACACTCTAAACATGACTGTGCACACATATGTGTTTGTGTGTGTTTGTGTGTGTGAGTCTGTGTGTGCACTCTATATCTATGAGTGTCCATATATGCATAGATACTATTCGACTCGTTCATTGCCATCACTCAAATTTAAAGGCGGCGCCTCTGGCAAATTGGAGGCTCCAACTCCAACTCCAACTTCGACTTCAACTTCAACTCCATCTTCAGCCACAGCTGCGCGTTGTACGCATGCGTAGACGGACACCGGCCAAATTCAATTATCGGTCTGTGCTCGTCTTTCGGTTCATGCTGCTGTTGCTGCTGCTGCTGCTGTTGCATTCAACACGCATACGCCAGGTGAACGGGCCAACGTTGCCAGGGCTCGTTGGGGTCGCAATTAAAGCAAACGCACCACAGCTCGCTAATTAATTTGCATTTGCGCCAGACAACTCGGGGCAAACTTGTTGATTACCAAACACACACACACATGCAAATATAATATAAATATACACACACATACATATGTATATGCCACATCTGCTACTTTGTCTTGTCAAGTAGCCACTTTGTGCGCACTGGCAGTTTTATCGAGTTAAGATTTATCACATTTTCCAATCATTAGTTGGGTTAGCTTTCATCACATTGAAACCCACATGGAAATATTAATTTGATGAGTTATTAAAGTCTGTATTAGGAGCAAGTACTGGTGATTACTAAATATTCAGGTATCATTTATGCTGTCTAATGACTGCATATAGCATGTGATCTTATCTGCTGATTGGAAGCAGTACAACTCAATCTCTGACTCGCTGAGAACTTTTTATGATAATTGCTCTCATTATCTTAACAGCCTTTAACAAAGAGAGAAAGCAAAACTTATAAAGTGCGAGAATTAAATAAAATGTATCTTAAACATAACCGTTGATATAACCATGATTTCCAAGTATTGCACAATTAGACTCGCTAATCAAGTAATAAAACAACAAGTCAGAATGGCCTACTCAAGACAACTACTCGACTAGCAAATACCCTGATATCAGTTCCATTCCCTTACAATTGTGTCTTTGATTTCTATCGGAAATCGATTTGTATCGATGATAACGAAACAGCAAAGTTATCGATAATATGTAATATAATTGATCTTCGTGAGTTAAACGAGAACTTTTATTCAACATTTGAAGTGTCTGAGATCGACTCTTGCATCCAGTGGAAATCGACTCGGCTATGGAAACTGATCGGGAATATCTATAGATAGATGTGTAGATGATTCCGGTTGCCTGTTACATACCTTAGCACAAATTCGTTATAACCATTTTGCCATTTTTAATGGATGCAGTGTAGAAATTGAGTCTTCAAGATATTTATTTTATTGCAACTATTCATTTAATCATATTAAATGAATGCATAAAAAAAAACTACTAAGCTTAGTTTCAGACGATCTTCGTTTCCGTGAGTGCTTCGGCTTTAGAGCAATCGAGTTTATTTTTAGCATTTAACTCGGTTTTTCATCTAATTAGAAGTTGTGAATTCTCCATCTAGACCGAGGTCTGAGGGGGCACAGTCGGAGTCTGAAGACTTTTCGCGTTTTCCTTTTGCGCAACGGGAAGTGACTTTTGTTTTTTGTGTTTGTTTGTGTGTGTGTGTTTTTTTTTTATAATATTTATGCCGCCGGTTGTTATTGTTGTTGTTTTAGTTGATGTTGTGGTTGTTGTTGTTGTTGTTGGGCGTTTGTATTTAAAAGAAGCAAAACTTACGGGCGATAAAAATGTTGCAACAGCAACAACAACATCGGCGGATTGATGGCGAATAAGAAATGGCGAAACAGCGAAAATGGCAAACTGGCAATAAGAAAGGGAGAGGCAAGTAAGAAACTTGAGCTTGGCGTGTAAACGGTGCTTTTTTTATCTGTTTTTCTTCGGCAAACCTTCTGATGTGGACAGCCGGCGACGCCGACTGGGTGGCCAGGCCAGCAGCGTGTACCCACCGTGAAAACCCAGACTTGCCCCACTCCCCACATTGCCCACCTTTTCCTCCACTTTTCTCTGACCACCACCGGGTTCGAGTGACGTTGCGTCTCTGAACTTGGCTAAGGGCGCTTCAGTTGCGAGACAAATGGAAATGAAGCGTTTATGCTGCTTTATTGCAGCGATAAGAAAAAAGAAAAAGAAAAAGAAAAAATGAGGCATTATTTTGTTGTTTCAAGCAAAAGTTTCTGCTTTACGATTTGCGTTACACAAATACTTTCGCATTCTTATTTATATGTATTTCTATATTTCCCATTTTTGCTTGGGGCACGGAAATGCCGTGATGCGAACTGGTCACAGGTTGAGCTTTTGCGGTACTCGTCCTCGTCCTCGTCTTCGTCCTCGTGCTCGTTCGTGGAATGCGGCAATTTCCTCGGCCGTCTCCGGCCTTAGAAATATTTGCAGTGGACGTTCCATGGACACTGAGCTGGCCATGAGTCCGCGGTTTTCTTATCCCAGTGGGATATCTGAGTCCTGTGACCGTGGCAGCAAAAATGATTCTGGCATTTAATTTCCAGTTTCAACGGTGCAGGAATTACGTTGCGTTTTAACAGCTTATTTAATGCGAATCAAATCGAGCAGAAAAATCATTCTTTTTCTTTTTAACTTTAAACAATGGCAATGACTTTGAAACACTAACCATTTGGCAAGTAATGCACAACTACAGCATTAAATTTCAATTTTGCATAGAATGGAATCTAATAAAACACAATTTAATATTTCAATTTTTACAAATGGGAGAAATAGGAAGAAATAGAGAACGAGACTTTAAATTTAGAAGTTGTTTTCAATTAATCGATGACTGCCTTCGTTTTGCGTATATCGATATAAATCGATTGGTAACAAATATAAACACAGTCATATTATTGATCAACTGCACAAATTTCATTAAAATCAAATAAGAAACACATGTTTTATTAACTAAAACATATTATATTCAACAAAATACTATTAATAAGATTTCTAACGTAGCTCTCTAAGCATTCCAAATTGTTCATTTAGTCGCTATACCAGCCGCAATATATTCAATATTTAATTGCTGTCGTAGTTCTAATTAGAGGCCTTCACATGGTAATTGACTACCGGGCAGTGTTATTTTGGGCAGCTTTGCTACTCATTCACCTATCATCAGCAATAAACCGTGAACGCAAACAGTAGCCGATGTGATCAGGCAGTTGAAATTCGCTTCTATTCGCTGCGATTGATCACAAAAGCCAATCAAATGTATGGCTCAAATATGTGTGCACCTACATATTACATACATAATGGAGAACAGCGTGAAGCCAATGCGATCAAGAAACCTCCAACGCCAAAAAGAGAAAAAAATATGTACAAAAATCACACATTTTCAAAAGCCAAAGCCGCATCATCAATTATGCTCAAAGTTGCTCAATGGCAGCCCAACAACAAAAACAACACACAAAGCTGTCAGCGCGCGTTGATCTGTCAAATGCCGCAACCGAAACTGAGACCGTGGCCAAAAGAAATGCGGCAGCCGCATCCGTAACTGAGGCACCCGCCGCCGGCACCGTTCGTAGTCTCCACTTGGCGACGCTTTTGTTGCATTTCCAAACAAAATATTGATTAATCGTGACGGTCGGCCATTGCTTGCTTGCCACTGCCACTGAATGCCAGCCAAGTCTACTTGCCAGTTTCATTCAAAGAGTTGTCCAAACGATCCTAAGCTGATTTTTGGCCTTCAGCTGCGCACTGAACTTGAGTCCGCATGTTTTTTTTTTTGTCTGTCTTTGTCTGGACAACACCACACAATATACGTTGATAATTTTTCACAGTCTGGTCTCTTTTCACTCCATAAGCCGGCGGCACTTTTTGTCCATCGATCGCTATTGTTAAACGCCACCCCCGCCCCCCCAGATACACACACCATCACATTGCGAAAAGCTGCTCAGCTTTTCATGGAATTTTTGCCACGCTTGAAACTATAGTTTGAAATGGTGGATGATGACAGCAAATACTTCTCCATACCTGTATAATTTTTGTCCACAATCGCATTTGCCGGAGTCACATAATTTGTTGTCACCTTTGGCGATCTGCGATTCGAATTGGAGCCCAAAGGGCGCCGACGGTGACAACTTTTAAATTGGCATTAATTTTGCATTGATGGTGGGGACTTTAATTTTCATTTTTAATTCGCTTTCAATCTGGCAAACATGCTGAGAATAAGCATAATCCAGATCCACCCCAGCCAGAGAGAGTCGGCCATGCACAAAAAAAAAATAAAAAAAAAATAAAAAACAGAAAAGGGAAATGTAAATTTCTTCTTGTTGTTGAGCGTTGAAAAAGTTTCCCTCGCTGATTTTGATTGACAGCTCTTAGCATCCCTGATGGATTTCATATTTGAGCCATGGCTGAGGCAATACTCGCAGCACCAGGCCAAAATGACAGACCTGACACACACACACTCCATGGCTGGGGAGGGGGAGAGGTCTATGGACCAGAGGCTTAGGCTCTGCATAAAAGTTTACCAACAGCTTTCATATTTCCCTGACGAGACGAGATGAGACGAGACGTTTCTTTCTTTCTTTTTTAGACACATGCGCATCTGTATATGGTATATATAAATAAATATGAAAATATGCTATATACGAATACATAAGGCGCTATACGTTATACATTGTACATAACGAGAAATACCTAGTTGTGGAGGCCATGTTGAAAGTTTTTCAATTTGAGCCGCTTTAAAGTATTCAACTTGATTCAATGACGATTTGACACGAGCATCGCTCGAAGTGGGTGTTTGGTTCAGTAGAAAAACTTTTTTTGTTTTTGTGTAGATATCATGGAAAAAATGTGACCATATTTAAACGGATAGCAGATATTTTTGTTGTTATTGTTGTTGTGTTTCTTTAGCTCCCATAAGCTGCCTAATGGCTTTTTGATTTTGGCGGCCAACAGAAAATAAATATTATGCTTGATTTTTTTTTTTTAATATAAATTGAATTCTTTTTGTTCCATTACAATTCAAACATAATTAAACTTTTATTCCCATTAAAATAGGGAAAATTACGGAGGTATGTCTGACCACAAAAATATATATTCTTGATCAGAACAGCTCGACTCTATATCTATGCTGATCCCAGAAACTATAAGCGCTAGAGACTTTAAATTTTGAATATAAGTTAAATAAAGCATTGAGCCGATTAAGTTAATTTAGTTTTATTGATACAATTGTATGTTTTCTACATATTTTGGACCCACTGTAAGAATGTCATTAAAATCGAATAAAAAACACAGATTTTTTTAACCAACTTATATATACATATACACTCAGGGTATCTCCAGTTCGAGCTGTCTCACCTAGAGCATTTTAAATCGTTATTTAATATCAGCCAATTTTAATGTTTTAATTAATTCAACCTGTATTCATAGCGATTAAACTATTAAAACTAACCGAAATAATATTTGAATGCATAACAAAGTCAACCTGCACCTTTAAAATATGCACTTTTAACAATGACATTTCAAAACCATTTCATTAATCAACAATTTTCACACCTACCTCTCACTGTGGCAGCAACAACAGTAACAACAATAAGGAGGGTCCTCCGAACAGAAGGAATGCCCATAGTTTTGTTATCATTGTTTGTGTTGTTTGTGTTGTTGTTGTTGTGAGTGCTCTGTAAATGTTCTGTTTGTTGACATTTGCTGGTCATGAAAAGTCATTAAATATATTTCTCTAATTTATCTTTGCAACTGGCAGCTCGGCAAAAGCATTCAAGTAGAACTCCAACAACCCAACCCACCGTTGTGGGTAGGGGTATTCGTAATCGTAATCGCACTGTTTTGTTGTTATTGTTGTGGCTGTCGCCAACAACCCTTTCGTCCTGGGCTAATTAAAACTAATCCAGATCCAGAGCAAATGCTGGATCCTTGAATGTAGTGCTGTACACTTTTCGCATGAATGTCAATCAAGCCCACAATTAAATGATTGAAGACATTCATCTGGCCCTCAACTTGGACTGTCTCCCGCTTGGCAGCTAATCAGCAGGCACTCAAGTGCCGGACTTGGCCATCAGGCAAGCAACAAGCTTTGAGCCACGTTTGTCTTTAATGCGATTCGAGTAGCCAATAAAATAGATACAACTTTGTATGAGGCTCCAATTTAAAATGCCACTTTTTTGTGTTTTTCTTAGAGACGAATAGCAAATAGATAGAGATACAAAATATATATTTTCATGAGAAATTGACTTTTTCGACTGGCACATCGTCGTCATCGCCTTCTTTTCCGTCTCGGTCGTTGTCTTCCTTTGGCTGGAATGATTAATGATGCATCTCTGCGCATTCGTACGTCTTACGACCCACACCATACTACACCACACCACACCACACCACACCAGAGCTCCCATTGCCTCAGCTCTGTCTGCATTTGTATCTGTATCTGTATCTGTAGCTGTAGCTTGTGTATCTTTTTGTTGTCAGCTTGAGCATTTTTACTGCATCACATCGCATCGCATCGCATCCTAGCATTTGGCATTTTGGAGAAGCTGAAGTTGGAAGCCGTAAGCTGTACATCCGCTGTGGCTCCTGCTTCGTCTTTGACTCCCATTGTTCTCCCATTTTGCTGCCTTATTTGATTGAATTTGTCAAGGCACTTGCTGTGCCCCCTGAAAGTCCTTTAAAGTTCTGTTAGCCAAGGCAAACATAATTCAAGCCATTTCCCACCCATGTTTAGTCGCTGCAAAAATTTACAAGATAAATTGAGGTCATTTTTTTTTCTCTGAGTTTTGTTTGTCCACCTTTGGTCAACTTGTTTGCGGTTCTTTGCTGGCGGCGTTCAAAGGTCCTGCCACAAATTGTGTCCACCTTGTTGACCGCTGTCCCGTTCATGTGTCCACAAATGTCCGTAATGTGCATCCCCCATTGACTAGCATGTGTAAGTGGTCAAGCACATAACCCAAACCCAAGACACACTCAACTTATGCAGAGTGGTATTCGAGGAAAGGCAGCCAGGGTTGTCCAGTAAAAAGCTGCATTCCTTGAAATGGGGCTTGAGAATGAATACACTGTCAGACTTTAAAATGTTATAATTGCTGTTTGTTGGTTCAATTACCCATTGCATTCTATGTATATATACTTATAGTTCCAGAATAGAATGTGCGATAGTCTCCATTGTGAATCGAGCTTTGCCTAGCCGCACCTCATATTTAAATATATTGTTAAGCAGGTTTACAGTAGATTCAAATGAAACAAGACAAAATATCGAGTTGAGACAACTCGACTAGGAGATACCCTGAACGCAGATTTTAAATAAGTTTTGCCTAACAAAATGTAATGAAATTTTTGTAATATCTTTATAAACATATCATATTCTGTATTAATTTGATGATTGATTTTTACAATTGTATCTTTTTTGATGAATATGAAGAATGTATATGCTTTTAGAGGGTCGGACATGCTTGCTTTTGTACACTTCATTAAATATTTTCCTCTTTTTTAATGGGTTAATATTAATGTTAATATTAATATTATTAATTAGCTGGACAACGAAAGCTCGCAAGTCTAAGTGCAAATTTAATTATTTACTTTTAATAGGCAATAATAATCTGATAAAAATGCTAAGGTTTGCTAGACAACTATATGAAATATGTGAATTTAATTTCTTGCTGTGTAGTCATTTTAATAAGCAATAAAAAACTTACAAATGCTAGTGTTACCTTGGGTGCGATGGAAAACATTTTGCAACAATTGCAAAATTTACGCTCATTTTAATTTCGTTGTTGGCCAGGGGCAGCAGATGCGTTAAGTGTGCCTGAAGCCTGGAAAATTTTTTTTTAGCTTGGACCAACCAAAATGCAGACATAAATTTTCACATAGTTGAGTGCTGGGAGGAGGACAAAGTGCAACCACAAAAAAAGGTCCGACTGGACCCAAAGGGGCAGCGAGTGCGTTTAAGTGTTAACATGTAAATTATGCTCTCGGCGCTGTTAATAAGTGTATTCCAAGTGCCATAAATCAGTAGAAGCACTTCCGAGCTGAAAATTAATATTATATGGCCAAAATAGTATGCGACACACATATATTGGCCTAAAGGAAATTCCGTTGCTCTCTTGTTTATAAAACACAAGTGCAAATCTTCAGTTCAGGCAACCAGGCCAAAATGCTGCTGCACAAAGTATCCGACGTAGTTGCTGATAAGTTTCGTTTCGTTTCGACTTTTCCGGCATTTCATCCATGCTGATGACATTTCCAATGCACTGCCGACCCACCCCCACCCATCCCTAACGCCCCTCAAAAATTCATTTTGTAATTTTCAGATACCTTGAATGCCCTGCCAGCTGTCGTTCTGTCGTTGTTTGGCTCGGCCCTGTGCTTTCATTTTGACCAGGCTGCAGAAATTGCAACCAACTTGCTTTCATTTTGCTCCAATCTTATACAAGGAGGAAGCCTCCACTAACTCCCTGCCCTTTCACCCCTTCCACCACCAGCCTGTCTCCCTGCTTGTGTGTGTCTGCACGGTTTGGTTTAGAAAATTATCTTTCTGCTCGTCGTCGCCAAAAAGGAAATCATATTTTCTGCTTTTGTGCACTTTACTTTTCAGTTTCGCCCCCTCCCCCCCCCTCCACTCTCCCTCTCTCTTCTCCGGCATCCTTTGCCACTTGCTCGTAAACAGTTTGGTTGCAACTTTTGTGCGAAATTTATGCTTTTCGTACCACACTCTTGCTCTCTCCCTCTCTCGCTCTCTCTCTCTCTATATCTATCTCTCTTGCTCCCTCTGTCGTATCTGGGCACTTTTCGCAAGTACTGTTTACATGCGACTTGTTTCCACCGCCCGCAACGCCCACAAACTCCCGGTTCGTGTTTACCGGTGTATCCCTGTGCCCTTCTATTTTGGGCAAATGGGTTGCTCTCAACAGAAGTAAGTGTGTGTATGTGTGTGTGTCTGTGTGTGTGTGTGTGTGTGTGGCATCCCAACCAGGTGGTGTTTATGGCATCCGCTCTGCGAGTATTGATGAAGGAACTCGCATCTGTAATTCAATCAGTGTCTATGTCTGGCTGCCATTTGCTGGCTATTGAGACTCACTCCGTTCACGCAACTCCTCCACTCAACTGGAAATATTCTCGTGGAAACATTTTCCTTCTTCTTTTTTTTTGTGGCGAACATTTTCCATCGCATCAATGCAAATAAGGTGTGCGCCGCTAAAATATCAAATGCTCACTTTACCATAATATAATGATATGCGAATATTTTATTTTTGCTTTTTTAATACGCGCACGTGTGGTTTCTATGTTGTTGTTGTTGTTTTTTGTGTTACTTGTTTGACTCAAAGCATTTCAGAGAGGAAATTTATTTGCAACTATCTGAAGATATATTTTATTATGGCTATGAACTGAAGATCCTGCTGGAGATAGATTGCTGTAGAAGCAATAAAACACAAGAACATTATGGTATCAGTTTGACTTTACTGGGATATTATTAAGGTGTTATGGCATCTTAATTAAAGAAAATAAATAAACATATGCGAATTTTCAACCAAAGACTTAAAAGAGTATATTAACGGACAAAATGTAAGCTATTGTCTTTAAAGAACTCTTTTCGATTTTCAAGCATTTCGAATTTTTGAGAATTTAAAAACAAGTAAAAATGATGTAGTCGAGTTAGCTCGACTACGATATACCCTTAACCAAGTTGTTAAATATATTCTAGTTTTATTGGCAAATTTCCCTTTAAGATAAATTACACTCAAATGTTCAATTTTGCAAGTGCATTTTTTACAGGGTATTTTTACTCACAAGCCCGAACTGACTCGAAATGGCTGTCTACAAACGCTTGCATATACATACATACATACATACACACATACAGACATAGTAGTTATCGGGGCTCTGCAGAATACACACAAATATATTGACGCTTGCGTCGATTATCATGCCATCTCTTTCTCACACATGCGCTCGCCTATTTATGCATACAGAAAATGCTTGCGGCTGCTCACAGCAAGAAGATCAAAATAAAAATATATTTTCGACTATAACTTTGGTGTTTATTATCCGATCTTAATTTTGCAATTGATCAATAATGTAATACATATGCATGCATAATTTAGAAGCCTTTGCCTCTTAAATTGTGGAATTTATTGCTATCGGAAATCGAAATGTATCGATAATTCAGAAACTGAGGCAGTTATCGATAAAATAAAGCAAACTTGATCTGCGCCTGGTACACGAGAACTTATATTCCAAATTTGAAGTCGCTAGTTCTTATAGTCTCTGAGTGTTCCGCGTGTTCATACATACGGACAGACGGACGGACGGACAGACGGACAGGGCTATATCGACTCGGCTGTTGATCCTGATCAAGAATATATATACTTTATAGGGTCGGAGATGCTTCCTTCTGCCTGTTACATACATTTGCACAAAACCATTATACCCTTTCTACCCATTTTTAATGTACAAGATTCTAAGATTGTACAAGGAAGAACTACAATCTTAGGCACGCCCAGTAGATCGAATGCCGACTTCTAATTTTGCTTTACACATTTGCAAAACTGACCAATCGTTCCTAAGAAAGCTATATGATATATGTCAATATGTACTCCCTGCATGAATAATACGAATAATTTCCAAGTTTCATCTAGATAGCTTCAAAGTCGTTACATATTTTTGCACAGCACCATCTTTTTGTAATGGTTGTTCAGGGATTACAAATTAGAAACCAGAAAAGAGGCATCAATTGCATAATCAACGCATGTTGCTGTGTCCAAGTCAGATATTCAAATGAAGCCAGCCAGAGATGAAGAGGCAAACAGTAGAAGAAGCAGAAGAAGAAGAAGAAGAGGCACAAACAGTAGCCAAACTACTTGAAGTGTCGCTGGGACGCAACGCGTCAAGCCACACAGATTGATTGATTAGGTTTTTTTGTTAATTACTTAACGCTCGAGATTGGAAGCTGACTAGCGATGAGCTCGTGATGCGAGACGCTCGACAAAACTTGCGAGAGATGCGTTACCCGCATTTTGGTGGCAGTTTCAGCGACATCAGCTGCAGCAGCAGCAACAGCAGCAACAGCAGCAGCAGCAACATTGGGCTAGGTGAAAGAATAAAAGTTGCTGCTGTTGTGTGCAGTTGGCCGGGAGCTGAATTAGTTATGGCCGGTGACGAGCTGATGGATTGATTACAATCGCGAGCACAGAAGCTGAGCTCAAGTCACTATGCAACATCAGTTGCATTAGTTGCTTCTTCAATTTGCCGCAATATATCAGTGCGAAGGACTCTGATTCCTGTCAAGCCAGCAAGTTTCTGTTGTTGTTGGTAACGCCCCGAACCCAACTGTCGAACTCAAGCGTATGCAATTTGTTTTGTTGAAGGCGTTACGTACGCATTTGCCTGCATTTGGAGCTGTGCGCCATGTTCAAATTCCCATTTGCTCAAGACTCAAACAAGAAATGCAATTTTCAATTGCAGCTGAATTCGTTCCAGAAAACGGCCTGCCCCGACGACTGCGCACCGCGTACACAAACACGCAGCTGCTGGAGCTGGAGAAGGAATTCCATTTCAATAAATATTTATGCCGCCCAAGGAGAATTGAAATAGCAGCCAGCTTGGATCTGACCGAGCGGCAGGTGAGTGCAAGCTTTGACTCCTTCAATATACCCATTCCAATGCGACGATTTGTCTGTCAGTTGCCAGTCTGTGAGTCTGTCAGTCAGTCTGTCAGTCTGTCAGTTCCACCTGCTCTGCGCAAAAGGTGGAAGAATTGCATTATGTGTGATAAATTGCAGGCCGACAAGGTTGACAGGGCCTGATGCGGCTGAGCTGTTTTCTTACTTTACTTTAAACATAAAGTAAATATATATATATACATATATATATATATATACATATATATAAATAAATACATATATATAATAATACATAAGATGCGGCTGAGATGTTTTGTTACTTTACTATTTATCTAGATTAACACATTATGTAATTATGTAATGCAATTGTGGCTGCTACCATTTTGCTACCACTTCCAGGTTAAGGTCTGGTTTCAAAATCGCCGCATGAAACACAAGCGACAAACGCTCTCCAAGACAGACGATGAGGACAACAAGGACAGCCTCAAAGGTGACGACGATCAATCCGACAGCAGTAAGTGCATCATTCATTTTGAACATGGCTCAAGTGAGATTTTTCCTCAACCTAAGTAAAGTTTAACTTACTTTTTTAAAATGTATCGAAGAGTTAAAGCTGTTGTTGCGATCTTACACGAAGCCCATGCTTAAGATTTAAGTTATAAAAAAAAGCAAAGTTCTTTTTAGTAATTCCTTAGACATAGTGAAGGTGTTTTGTGTGTTAATGTTAAATGCAATCAAGAGAAAATTCCAGATGTACTCACTTCTTATTCAGTTTTCTCATTTGGCACAGCAAAATTAATTCAATTCACACTTCTTTAAATAAAAAAATAAGAAACATGAGGGGAAATTCATTCGCTCTTAAGACTTTTAATCTTTGCATCGCTTAGCTACGACTCATAAGATATTAATTTTTTCACTTCAACCATAACAAAAAAAAAGTACAATTCAAGTTTATTAGAAGCACTTTAGTAAAACTCTTTAAATACATTGAGCTATTTGGGAGCTATCATAGTTTTTCAAGATATGAAAGTTCTGTTTTTGCTGGCAAAAAAATAGCAACAGGGAAATGAACGCAGTTTTCAATTGAACAGAAAACACTTTATTAACAATTACAGGGCGATTCTATAATATTGGAAACTGTGCTTGCACATTCTGTGCATGAAATTAACACAATTTAATTATTATTATTAATATGCGCGTGTCATTTTAATTGTTTAAATAATCGGATGATCAACGTCCAACGAGATGTCGATGCGCAATTTGCGCCGGTTACAGATATTCTGCCACTTGTGGAAGCCAATGATCTCTCCCGAATCCTTTTTAGTTACTAATCGCCAGTTTCATGTTTACTTGTGTGTTAATGTTATCGTTATCTTTATCGTTATCGATATCGTTTTCTTTCCTTTTCGCATTTATGCGGAGAGTATTTAACAAGACAAATATTTACACATCGGTTGTAATCGCACACAATATCTATATATATTTAATTGCTGATGTAGATTCTAAATGCCTTAAGTTTTGAAGCATTGTTTCGATCAGTTAACAACATTTAAATCTCTCTATCCTATTAGTGCTGCTTGGTCTCTCTTTTTCCCTAAAACGCTCTTGAGAAATATATACCTTACGTGAATGTGACTCTTTTTCTAAATTTATTCACAAAAACGGACATTACAAGATGTGGTTTGAATGTAGGTCATCTTAAAGCGTCATCAAATTGGTTACAAGACTTGCGGCAACACTGTCCCTTGCTTGAGAGAACGTGGAAACACTTAGCGCGAACCACACTCTCAGTTATCTTTGGCGGGTCACTTTTACTTTTCGAGGCACATTTGCGCCAAGTTAGAGAAGAGTAGAATATGCTGTTGCTTATCAATCATTGTCAGATGGAGGAGGGCTCAACTTTTGCATTTCGCTGGGACTCAAGAGTTTCCCGTCTACTCACGCGACTGTCTGCTCGAGGACTGCTAACCCCTTTTCGTGATATCTTCAGCTTTAATTCGGTTTAATTCGTCTTACTTTGGATTTGGGTTTGGTCTGGTTTGGTTTTGGTTTCCTTTTGGTTTCCTTTTTGAAAGCAAATCATTAATTTTTGTACTCATACTAATTCCGACTGATTTACTTTGGTTCCCGTGAAATGCAGACTCCAATTCGAAGAAATCGTGTCAAGGCTGCGAGCTGCCCTCCGATGATATACCGGACTCCACGTCCAACTCGAGAGGACACAATAACAACACGCCCAGCGCCACAAATAACAATCCGAGCGCAGGAAGTCGTAAGTGTTTAGATTGCCATTGCGTAATCATACGCATCCCTAACAATTCAATCAATCTAATCTAATGACAGTCACTCCGAACTCATCACTGGAGACTGGTATCTCGTCCAATCTGCTGGGCAGCACCACCGTATCCGCCTCGAATGTCATCAGTGCCGACTCTAGCGTCGCGTCCAGTGTCAGCCTCGACGAGGACATCGACGAGAGTCCCATCAAGGTCAAGAAGAAAGACGACACTCACGGCCAGGTAAATTTCCAATATACTTCATGTGTACCAGTTAATATATGTATACGATTAAAAACTCAAGGTGATTAAAAAGGAGGCTGTTTCCACCTCGTCCAAGGCCTCACCCTTCGGCTACACCGACGCGGGCCCCAGTTTGGCCAGTTTCCGGCGCGATTCCGATGCCTCTGTTGCCTCGAATCCGCCCGTATCCAAGGCCGGCAACAAGAAACGGTATCAGAATCCAAACGCGAATGCTAATCCAATTGGAATTGCGTCGCCACTGAGCGAGAGCAGCAACGCTGCTGGGCCAGCTGGTTATTTTCCTGGTGCTGGCTACTATCCAAATCCGAATGTCAATCCGGGTGCAAAAGCATTGCAGGCGCCGCAGCAAATGCCGCAGGATTATTATGGCAAATACGATATTGAGTTTGCAGCCTCGCCACACCACAACCCGCACAAGCAACAGCAGCAGCAGCAACAACCGCTTCACGGCGAATATCTAAGTCCCAAACCAAACACCAATGCCGCCAACAGTAATTTCCATCAAAACAGTCAACAGCAACAACATCAACATGAACAGCAGTTCTACTACAACTACAACGACACCAATGGCGGTAGTGCGTACATGAACCACCAACAGCACCAGCAGCAGCATCATCCAGTTGGTGACTTTGAGGCGCCACCTATCAACGGACCGACGAACTTTTATGATCCCAAGTCGCAAACCAGCGGCGCTTACTACGACAACATGAATTTCCAACACCAACACCAGTCGGTTGGGTTCCAGCAACAGCACCAACAACAACACCAGCAACAGCAGACGCCAATAAATCACCAACAGTAAGTACAAATTCAACGATATGGAGTGTAAAACGACATCTATGTACTGCTGCTGCAAAACAATGCTCCACACTTAACGAAAGTCGAGACATTTACTTTTTGCTGCACATGGTTTCGTTAAGCGAAAAAAAACTTGTTCGATTTAACATATTTTGCTATTTCATTTATTTTTACACAGGCACATGCATCACATTGGAGCCGGTGAAACGTACAGTGCGCTCGGTCTGCAAATGGAGAACTGCGAGAACTACAACAACTTTGGCGGCGGCTACTATGAACCGGGCGCTGCTCAGCAACAGCAACAACAACCGCCTGGGCCGCCCACTCACACCCATCCCCACCCTCATCCGCATCCACATCCCCATCATCCGCATCCGCATCCTCATCATATGCAGGCGCAGGCGCATCCACATCTACATGCATCGCACCACAATCCAGTTGCGACTGCAGCCACTGTCCAAGTGGTGGGCGGAGGAGCGCCCCCGCCACCCACCTCTCATGTCCACATACCAAATGCAAATGCGGCGAATTCCAATTTCGTGATGAACGGGGGTGGTGCGCCTGTGGTGACTGGTGGACAGATACAGGCGTTTGCCAACACTGGTGGTGGTGGTGGTGTTGCGGCAGCGGCTGCCATCAGCGGGCTGGAGAATTCGAACAGCTCGTCGGACTTTAATTTTCTGAGCAATCTGGCCAACGATTTTGCGCCCGAATATTATCAGCTAAGTTAGTTTTGGTAGTTTGTAGACTGTAAATAAATTGAAATCAAAATCGATGTAGTCGAATTTGTATGTGTGTGTGTCAGCACTTTGTCGTAGCACATATCTGTAAATATGTTTGTATGTCTAAAGGATATATATATAAATATATATATAGTAGGTAGAGTGATAAATGAAATATGCATTAATCTATACTTATTTCTCACTTTGAGACAGACATCTAGTTTAGGGCTCTCCTGGAAAATTTTAACTTTGGCTTAAAGCCAAAACACACACACAGACAAGATGCAAGTAAATTCTCTCCGTTCGAAAATTATCTAAAACTGTTCAAATTGTATATAGCTAGTAAAATTAAACATACTTCAACGTAAATTTCCACCAGTCCTAATCAAAAGTAATTCAACATAAGTCTACTATATATATAAATCGAATCTTACTTAATGGCATACAGAAATCAAAAATATAATTGTGTTTATTGTAAATATTCGAAAGTAGCTCAAATTAATATGTGAAATTTACATATTGAGTCTGTTTGCGGTTCAAATAAAATTTCCATTATGTAATATTATGATTAAAAATTATATTCTCATGAAATAAAATAAATAACTCGTAAATGAAATGATAGAGAAAAATCAATCAAATTCAATGTTTGTAATGTTTGTATGTTGTAAATGTAAATTCCAAATACAATTAACTTTAAAAACATTTAGCCACTAAATAATGAACGAATGAAATAAATGAATAAATATTAACCAATTGTCGTTGTAAGCTGCAAATTACATTAATTAACGATAAATACGATAGCAATCAAAAACTTACAAAATTAACAAAACAATTTCTTATCGCAATAGTTTTTGCTCAATGCTTTGCTGCCTCCTCTTTTTTCTTAATACCCAGCTTTGGAAAGAAACAGAAATGTTTTCTGGACAGGCACAAAAAATTCGAAATCCGACTGACAATTAATGATGGATGAGTTTCGGGTGTTGAAGGGTACCAAACCAAATTTACCGTTCTGAATAATGTGTGTGTGTGCGTGTGTGTGTGAGATTATTTTGAGCATATGGAAATAATTTTAAACTTATGAATACAGTTTGCAAGAATCGGATGCCGCATTGGGTTTTTATCTAAAATGATGTTCCTCAAATTTCAAATATGTGTATTTAAGTAAACATTTACATATGTTACAAGTTAAATACTCAACAATTCAACAACTATTATTCAGAGTCTTCAAATTATGGTGATTTTATTTCATCTATTTGCAATTCTATAATCAAAAGATCTCGACTAGCAGGTATCCTGAACCCAGCTATCATAAATTATAATAAAATTCTTCGTGATAGAAGTTTGTTCTTTGAAGTTTCAATATGAAAATACGTTTCTTTTTGAACCTGTGTTTCTTTGATCAATAATGCCTGTGTATAATATATATAAATTGGAAGGCTACTTACAATTGTGTGCTATCAATATAGTTAAATGCATTTTATCTGAGTGGAAGTCTATTTAAAACTCTAAAAATCTAATACACGGAATTAGTTAAATTGACTCGGCTTTTGATGTTGATTAAGAATATATCTACATTATTGGATCGATCACGCCTGTTCCATATTGTCTTAAAAATCCATGTACCCTTCTTAGATTTTTAATGGGTTCGGGGCATAAAAAGTAACATTAAAAACAATTCGATTGTTAATTTCGATACCCATTTTTAGGCTAGAATAACCAATATACACTTCTAAAACTGCAGACCAACTACAGAAAATCGATGATCTGTGCTTATTTCAAGAGAAATAATATTCCAATGATGCAACGCCTTAGAAAAGTTACCAATTGTATGCCAAGCAATACCATATTCAATAGTTGATTGAAATTTTGAAGTGCGGACATTTATGCTTCATTTGGTCATTGGTCGCAGTTTGTGTCCCTAAGGACTGCGACTGCGCCCATTCGTCAACCAGCAGCATTTTTTTTTTTTTTTGGTTTTGCTGTCAACATTTCAGATACTTGGATGCCACATGCTATTCTAGTCCCAAACCCTAAGCCGAACCCAATCCGAATCCCAGTCCCAGTCCCAGTCCCCTGTCGTCGGCTACGCACAATTCTGACAGAAGCGGTGAAAATTTAAGATTGATCACCCAGCCGAGTGTTTGTCAAAATTGCCTGCTGGCTGGAATGGCTGAGCTGCTGGGTATCCGTGTCCCACATCCGTGTCGAGTTCCGTGTCCATGTCGAGTACTGGCTAAAACCTAAACAATGTCACAACTGTCTGCTGTTTGCATGTGCCAGGGTCTGTATTATCAGTATCCCCAATGGATATATAGATTTATACACATATTTTTATTTAAACTGGTCAAAAGCGTGTCCTGCTCCATTTCATTAAATTACGGCACACACGACTTTGGCTAATGCCAAATGGCAAAAATATATTTTGCGAAATGAGACCACAAAACAGAGGTTAGCTAAAAATAACTGTTCGACTATTTGGCTAAGGTCAATGCTGTATAGACATTAATTAGACTTCTGAAATTATAAAAAAAAAGAGATTCGAGATATTTTATTGAAATTGATTTAAGGGGAGCTGAGGCCAACAAAACGTCAACACATTGGCTTTTGCTTTTGTAGCTAAGCTGAATATTTGTAGCTGGTATTTGCAGCTGGTTAATTACAATAAGTTACCATTAACGCTGTGTAAGACATTTTAAGAGCTACCGTCGTGTTGGCCACTTTAACAACGCCGGCCGATGACGAAGACGATGACGTTGACAAATATGTAACGAATATTTGAAAACGTTTAGTCAAAAATAGCTAAGGTTTAGGTTTATTCATATGTGAAATTTCAACAGTTACAACAAAATCAGGCAACTCGGGCTGTTGCCAACAAACCTTAACGGCCCTGTGCAGGCCAACATGCAACTGGCCAACAGGCCAACAAGTGAAATAATAACAATTAAAGTAATACAAACCAGTTTGCATTTAAGTAAGTTTGGCAGCACCACACACAACACACACACACACACACACACACGCACACTCACAGTGCTCAACACATGTGGTTTATTTCTTTTGATCCGTAA
